# Universal and versatile morphology engineering via hot fluorous solvent soaking for organic bulk heterojunction

**DOI:** 10.1038/s41467-020-19429-x

**Published:** 2020-11-04

**Authors:** Tong Shan, Yi Zhang, Yan Wang, Ziyi Xie, Qingyun Wei, Jinqiu Xu, Ming Zhang, Cheng Wang, Qinye Bao, Xin Wang, Chun-Chao Chen, Jingsong Huang, Qi Chen, Feng Liu, Liwei Chen, Hongliang Zhong

**Affiliations:** 1 School of Chemistry and Chemical Engineering, Frontiers Science Center for Transformative Molecules, and In-situ Center for Physical Sciences, Shanghai Jiao Tong University, Shanghai, 200240 China; 2 i-Lab, CAS Center for Excellence in Nanoscience, Suzhou Institute of Nano-Tech and Nano-Bionics, Chinese Academy of Sciences, Suzhou, 215123 China; 3 Key Laboratory of Polar Materials and Devices, Department of Electronic Science, School of Physics and Electronic Science, East China Normal University, Shanghai, 200241 China; 4 Bruker (Beijing) Scientific Technology Co., Ltd, Beijing, 100192 China; 5 School of Materials Science and Engineering, Shanghai Jiao Tong University, Shanghai, 200240 China; 6 Oxford Suzhou Centre for Advanced Research (OSCAR), University of Oxford, Suzhou, 215123 China

**Keywords:** Solar cells, Photovoltaics, Solar cells

## Abstract

After explosive growth of efficiency in organic solar cells (OSCs), achieving ideal morphology of bulk heterojunction remains crucial and challenging for advancing OSCs into consumer market. Herein, by utilizing the amphiphobic nature and temperature-dependent miscibility of fluorous solvent, hot fluorous solvent soaking method is developed to optimize the morphology with various donor/acceptor combinations including polymer/small-molecule, all-polymer and all-small-molecule systems. By immersing blend film into hot fluorous solvent which is utilized as liquid medium with better thermal conductivity, the molecular reorganization is accelerated. Furthermore, fluorous solvent can be miscible with the residue of chloroform and chloronaphthalene above upper critical solution temperature. This mixed solvent diffuses around inside the active layer and selectively promotes molecular reorganization, leading to optimized morphology. Compared to widely-used thermal annealing, this approach processed under mild conditions achieves superior photovoltaic performance, indicating the practicality and universality for morphological optimization in OSCs as well as other optoelectronic devices.

## Abstract (web summary)

Morphology control of bulk heterojunction organic solar cells has been a challenge for realising optimal photovoltaic performance. Here, the authors utilise amphiphobic nature and temperature-dependent miscibility of fluorous solvent to promote molecular reorganisation and morphological optimisation.

## Introduction

As one of the clean energy solutions, organic solar cells (OSCs) have attracted numerous attention due to the outstanding advantages of light weight, flexibility, and high-throughput fabrication^1–3^. In order to achieve high performance, it is distinctly essential for OSCs to efficiently convert the photon to free charge. Although the photon-to-charge conversion process is significantly complex, which involves several steps, such as exciton diffusion and dissociation, charge transfer and transport, and charge recombination and collection^4–6^, it is well known that the morphology of bulk heterojunction (BHJ) blend film plays a key role in all these processes^7–14^. Furthermore, the morphology is also tightly correlated with the device lifetime which is crucial for determining whether OSCs are able to get access into the consumer market^15–17^.

There is an urgent need to develop a universal strategy being applicable in varied material systems and devices. Since the solution-processing fabrication is too fast for organic molecules to assemble into the way as expected, additional post-treatments are usually needed by the employment of heat or solvent as driving force to reorganize donor and acceptor molecules^9,18,19^. The post-treatments, including thermal annealing (TA), solvent vapor annealing (SVA) and solvent soaking (SS), are commonly applicable for most of OSCs^8,9^. For example, almost all reported OSCs with power conversion efficiencies (PCEs) over 15% can gain further promotion in device performance by utilizing TA^20–31^. However, a high annealing temperature is often required to initiate the molecular reorganization in TA, therefore it is not suitable for polymer substrates of flexible device, which is considered as one of the most promising applications of OSCs^32^. In addition, TA is usually performed by heating the device on a hot plate, whereas the gaps existing in the interfaces of hot plate/substrate/buffer layer/active layer cause the uneven distribution of heat flow, consequently restraining the utilization for large-area devices. To utilize SVA, the fresh-made active layer is left in a solvent vapor atmosphere, thus the slow drying process of solvent residue in BHJ can allow the molecular reorganization to be more thorough^33^. Notably, SVA can be done at room temperature or relatively low temperature. However, the effect of SVA is highly determined by the vapor pressure and annealing time, whereas sometimes the change of only a few seconds will significantly vary the device performance, leading to poor practicality for scale-up manufacturing^8,9,25,34^. Regarding SS treatment, it is reported that the photovoltaic performance of some fullerene systems is somehow improved by spin-casting polar solvent on top of the active layer or immersing the blend film into polar solvent^35–38^. However, an effective SS treatment is currently lacking for non-fullerene based OSCs. Considering the implementation of TA, SVA, and SS, we speculated that the morphological optimization may receive the merits and overcome individual drawbacks of the existing post-treatments if both effects of heat and solvent were simultaneously utilized. Naturally, SS in hot solvent could be an ideal choice. By taking advantage of high thermal conductivity and sufficient contact between film and liquid atmosphere, the molecular reorganization can be accomplished quickly and completely. However, the choice of proper solvent for the soaking process is quite challenging. Hot organic solvents will rinse out the organic semiconductors, while polar solvents like alcohols might destroy the underlying buffer layer, e.g., PEDOT:PSS and ZnO, via the pinhole penetration and lateral infiltration.

In this work, a novel post-treatment namely hot fluorous solvent soaking (HFSS) is developed by immersing BHJ into hot fluorous solvent for short time, and consequently the photovoltaic performance of BHJ is appreciably improved. HFSS treatment is applicable in different donor-acceptor combinations including polymer/small-molecule, all-polymer and all-small molecule systems, verifying the generality of HFSS for morphology optimization. Further studies revealed the versatility of fluorous solvent during HFSS processing. Firstly, highly fluorinated solvents are usually immiscible with organic solvents, meanwhile most of the organic conjugated molecules and metal oxides are insoluble in these fluorous solvents even at relatively high temperature^39,40^. This amphiphobic feature exactly fulfills the purpose of hot SS for BHJ^41^, and ensures fluorous solvent can be utilized as the liquid medium to afford better thermal conduction. Secondly, the distinctive temperature-dependent miscibility of fluorous solvent, i.e., the miscibility of fluorous phase and organic phase being raised with elevating temperature and then the fluorous/organic biphase being miscible homogeneously at an upper critical solution temperature, plays a crucial role in the morphological adjustment. It is well known that the residue of original processing solvent and additive commonly exists in as-cast film and will cause the slow deterioration of morphology and decomposition of components^33^. However, the residue can be employed as a useful ingredient to modulate the molecular reorganization in HFSS. After penetrating the blend film, the hot fluorous solvent is able to be homogenous with residue and then the mixed solvent diffuses around inside the BHJ. Due to the tiny amount of residue which has good solubility to organic semiconductors, the molecular reorganization is accelerated with stronger intensity and the larger region, leading to enhanced crystallinity and phase separation. For example, a well-defined fiber-like topography is evident in PM6:Y6 blend film after HFSS^23,42^. Therefore, attributed to the synergistic effects of heat and solvent, BHJs with HFSS treatment achieved optimal morphology which were verified by the significant increase of fill factors (FFs). In addition, compared to widely used TA, HFSS was performed at relatively low temperature with shorter processing time while providing better PCEs. This work demonstrates HFSS is a robust method for morphology engineering and might be applicable for large-scale manufacturing.

## Results

### Photovoltaic properties

**Fig. 1:**
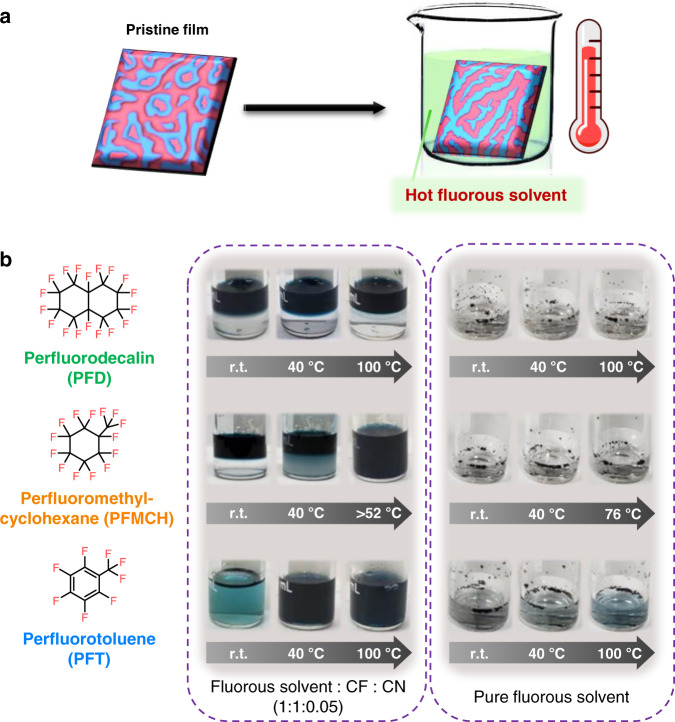
Schematic illustration and temperature-dependent miscibility of fluorous solvents. **a** Schematic illustration of the hot fluorous solvent soaking (HFSS) approach. **b** Chemical structures of three fluorous solvents. Temperature-dependent miscibility of fluorous solvents and chloroform (CF)/chloronaphthalene (CN) (v/v, 1:1:0.05) (Y6 as a color agent dissolved in chloroform) and solubility of Y6 in fluorous solvents.

**Fig. 2:**
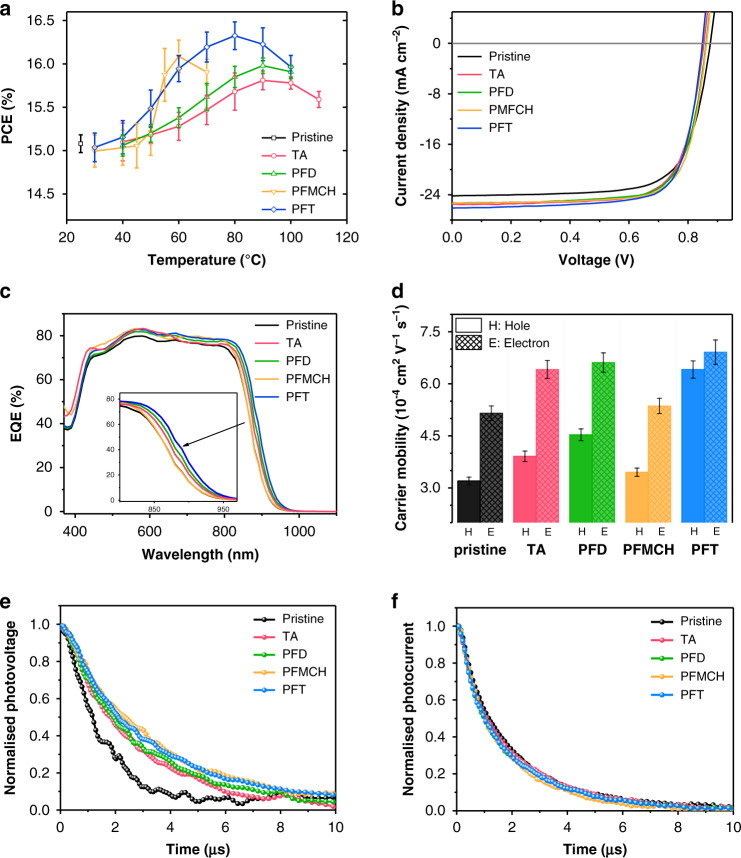
Photovoltaic parameters and charge carriers dynamics. **a** PCE versus temperature of post-treatments. Error bars represent one standard deviation from the mean (*n* = 20). **b** *J*–*V* and **c** EQE curves of optimized devices. **d** Carrier mobilities measured in single carrier diodes by fitting of SCLC model. Error bars represent one standard deviation from the mean (*n* = 8). **e** TPV, and **f** TPC of devices with different post-treatments at the optimized condition.

All the OSCs were fabricated with a conventional device architecture of indium tin oxide (ITO)/PEDOT:PSS (poly(3,4-ethylene dioxythiophene) polystyrene sulfonate)/BHJ/ZnO/Ag. PM6 and Y6 as a renowned combination are firstly utilized to fabricate BHJ by spin-cast from chloroform (CF) solution with the additive of 1-chloronaphthalene (CN)^43,44^. Figure 1a schematically illustrates the process of HFSS, wherein a fresh spin-cast film is immersed into a thermostatic fluorous solvent for a certain time, followed by the normal process of depositing electron transport layer and metal electrode. Details of the device fabrication and post-treatment approaches are described in the Methods section. The pristine device without post-treatment shows a PCE of 15.21% with a *V*_oc_ of 0.876 V, a *J*_sc_ of 24.18 mA cm^−2^ and a FF of 71.8%. Such high FF, which is tightly correlated with morphology, indicates the original device has already achieved excellent morphology. Although further enhancing FF and PCE may be challenging, it is more convincing to verify the effectiveness of HFSS on morphology optimization in this system. TA has been verified to be effective for improving the device performance in the previous literatures^23^, so devices post-treated by TA with varied temperature and processing time are fabricated as the counterpart. As displayed in Fig. 2a and Supplementary Table 1, the PCEs gradually increase with elevating temperature until around 90 °C, while a longer processing time (5 min) is beneficial for the PCE enhancement. As a result, the optimal device with TA realizes a PCE of 15.96% with increased *J*_sc_ and FF compared to the original device, which is consistent with previous reports^23,24^.

The fluorous solvent is initially designed as a better medium of thermal conduction to promote molecular reorganization. To exclude the influence from other factors, perfluorodecalin (PFD) is firstly used as the solvent for HFSS because PFD is immiscible with CF/CN (v/v, 1:0.05) mixture and insoluble to PM6 and Y6 at any temperature below its boiling point (Fig. 1b). After the treatment of PFD, the vary of PCEs versus treatment temperature (Fig. 2a and Supplementary Table 2) is similar to that of TA, yet a slightly higher PCE of 16.12% is obtained at 90 °C with shorter processing time (2 min) by this approach. This result verifies the hypothesis that PFD as liquid medium increases the efficiency of thermal conduction and allows the molecular reorganization to accomplish in shorter time, eventually demonstrating the superiority of HFSS compared to TA.

To further explore the merit of HFSS, the fluorous solvent alters to perfluoro(methylcyclohexane) (PFMCH) that possesses temperature-dependent miscibility with CF/CN. When heating to around 50 °C, a homogenous solution of PFMCH and CF/CN (v/v, 1:0.05) is observed as shown in Fig. 1c and Supplementary Fig. 1. Meanwhile, PM6 and Y6 are insoluble in PFMCH even at the boiling point (76 °C). At low temperature below 45 °C, the performance of devices with PFMCH treatment for 1 min is almost identical with those of PFD and TA (Supplementary Table 3). However, the slope of PCE versus temperature dramatically increases around 50 °C as shown in Fig. 2a and Supplementary Fig. 2. As a result, the highest PCE of 16.37% with a FF of 74.8% (Table 1) is obtained at 60 °C, while a PCE of 15.51% with a FF of 73.1% could be acquired by TA for 5 min at the same temperature. It is worthy of note that the threshold temperature at the turning point is in accordance with the upper critical solution temperature of PFMCH and CF/CN (around 52 °C). Thus, it is naturally speculated that PFMCH somehow works together with CF/CN residue to optimize the morphology. To confirm the existence of CF/CN, X-ray fluorescence spectroscopy (XRF), which has already been employed to successfully detect the residue of diioidooctane (DIO) in OSCs^45^, was performed. Supplementary Fig. 3 shows the wavelength dispersive XRF spectra of pristine and PFMCH treated films. The fresh-made pristine film exhibits an obvious atomic orbital transition of chlorine (2*θ* = 92.7°)^46,47^ while there is no measurable chlorine fluorescence in PFMCH treated film, indicating PFMCH has mixed well with CF/CN residue during HFSS and then the solvent mixture is fluxed away from the blend film. To further reveal the effect of PFMCH and CF/CN mixture on the improvement of device performance, a control experiment is performed by storing a fresh-made active layer in vacuum for 12 h, so that the CF/CN residue can be completely removed which is demonstrated by its XRF spectra (Supplementary Fig. 3). As shown in Supplementary Table 4, the CF/CN-free devices with/without PFMCH treatment show similar PCEs of 15.62% and 15.49%, respectively, demonstrating the mixed solvent of PFMCH and CF/CN is essential for achieving better PCE (16.37% for the device which is treated immediately by PFMCH after spin-cast). Notably, the device with vacuum treatment exhibits a slightly higher efficiency than the pristine sample (15.21%), which is might result from the effect of residue evaporation in vacuum on morphology modulation. Based on these results, it is concluded that PFMCH is only utilized as a medium of thermal conduction below 50 °C, leading to its effect is as like as TA and PFD. When the upper critical solution temperature is reached, the PFMCH penetrating into the blend film is completely miscible with the CF/CN residue to form a homogeneous mixture and diffuse around inside the blend film. Then, the photoactive component is selectively eroded and reorganized by the mixed solvent, which has slightly better solubility to PM6 and Y6 due to a tiny amount of CF/CN, eventually optimizing the morphology and enhancing the photovoltaic performance^48^.

**Table 1:** The Photovoltaic Parameters of Devices with Various Post-treatments under The Illumination of AM 1.5 G, 100 mW cm^−2^.

Post-treatments	*V*_oc_ (V)	*J*_sc_ (mA cm^−2^)	*J*_sc,cal_^a^ (mA cm^−2^)	FF (%)	PCE^b^ (%)	*µ*_hole_^c^ (cm^2^ V^−1^ s^−1^)	*µ*_electron_^c^ (cm^2^ V^−1^ s^−1^)
Pristine	0.876 (0.875 ± 0.002)	24.18 (23.92 ± 0.15)	23.56	71.8 (70.3 ± 1.1)	15.21 (15.08 ± 0.10)	3.34 × 10^−4^	5.40 × 10^−4^
TA 90 °C	0.854 (0.853 ± 0.002)	25.51 (25.22 ± 0.17)	24.73	73.3 (72.2 ± 0.7)	15.96 (15.81 ± 0.11)	4.11 × 10^−4^	6.73 × 10^−4^
PFD 90 °C	0.861 (0.860 ± 0.002)	25.30 (25.05 ± 0.16)	24.70	74.0 (72.7 ± 0.8)	16.12 (15.98 ± 0.09)	4.73 × 10^−4^	7.01 × 10^−4^
PFMCH 60 °C	0.863 (0.862 ± 0.002)	25.35 (25.11 ± 0.21)	24.56	74.8 (73.0 ± 1.3)	16.37 (16.09 ± 0.18)	3.59 × 10^−4^	5.63 × 10^−4^
PFT 80 °C	0.849 (0.847 ± 0.002)	26.09 (25.71 ± 0.25)	25.28	74.6 (73.0 ± 1.2)	16.52 (16.33 ± 0.16)	6.72 × 10^−4^	7.35 × 10^−4^

^a^The photocurrent *J*_sc_ values are calculated by integrating the EQE spectra;

^b^Average values with standard deviation in parentheses are statistically obtained from 20 cells.

^c^Measured in single carrier diodes by the fitting of SCLC model.

The success of PFMCH encourages us to take a step forward to choose perfluorotoluene (PFT) as the solvent for HFSS. As shown in Fig. 1c, PFT is miscible with CF/CN even at room temperature, while Y6 is sparingly soluble and PM6 is insoluble in PFT. With the extraordinary miscibility, the post-treatment of PFT is more efficient so that the optimized processing time is shortened to 0.5 min (Supplementary Table 5). Figure 2a depicts the curve of PCE versus temperature. Unlike the above two fluorous solvents, PFT can improve device performance even at low temperatures (≤50 °C). The PCEs with PFT treatment increase steadily from 30 to 80 °C, showing a bigger rising-rate compared to TA and PFD, while there is no evident turning point as appeared in the curve of PFMHC. The champion device was obtained at 80 °C, with a *V*_oc_ of 0.849 V, a *J*_sc_ of 26.09 mA cm^−2^, a FF of 74.6%, consequently providing the best PCE of 16.52%. This outstanding improvement can be explained by the idea that PFT is miscible with CF/CN residue at any temperature, and the mixed solvent promotes the reorganization of donor and acceptor molecules due to better solubility.

By taking a holistic view of the effect of HFSS on device performance, the post-treatment with fluorous solvent surpasses TA attributed to the enhancement of *J*_sc_ and FF, while HFSS is performed under the relatively mild condition with lower temperature and less processing time. The *J*_sc_ is further confirmed by the external quantum efficiency (EQE, Fig. 2c), whereas the enhancement mainly locates in the long-wavelength region. This is believed to originate from the bathochromic-shift absorption of the active layers (Supplementary Figs. 4–6). Compared to the pristine active layer, the maximum absorption peak by the treatment of TA, PFD, PFMCH, and PFT show red-shifts of 10, 14, 5, and 21 nm, respectively (Supplementary Fig. 6a), indicating different enhancements of packing order in blend films after post-treatment^49^. Notably, PFMCH processed at 60 °C causes a smaller red-shift than TA at 90 °C, suggesting both of fluorous solvent and high temperature are beneficial for the morphological modulation. The bathochromic-shift absorption of BHJ also rationalizes the slight decrease of *V*_oc_ after HFSS. Compared to TA, all fluorous solvents lead to higher FFs, implying the morphology of BHJ is more close to the ideal situation. In terms of these three fluorous solvents, the best PCEs raise in the sequence of PFD, PFMCH, and PFT, which is identical to the order of fluorous solvents’ miscibility with CF/CN. In addition, a turning point with a dramatically increased rising rate is evident at the upper critical solution temperature in the curve of PCE versus temperature in PFMCH case. These observations imply the homogenous mixture of fluorous solvent and CF/CN is crucial for molecular reorganization.

### Charge transport and recombination characterization

To clarify the effect of post-treatment on device performance, charge transport and charge recombination properties were studied^50,51^. The charge carrier mobility was evaluated by space charge-limited current method (Supplementary Fig. 8 and Supplementary Table 6). As shown in Fig. 2d, unbalanced charge transport, wherein the electron mobility is higher than hole mobility, is observed in pristine blend film, which might be rationalized by the idea that small-molecule Y6 has stronger crystallinity to favor the electron transporting. With the post-treatments either TA or HFSS, the electron and hole mobilities are simultaneously enhanced. Nevertheless unbalanced charge transport properties still remain except PFT case. PFT treatment provides the best charge mobilities of both electron (7.35 × 10^−4^ cm^2^ V^−1^ s^−1^) and hole (6.72 × 10^−4^ cm^2^ V^−1^  s^−1^), in particular the hole mobility is twice as high as that of pristine BHJ (3.34 × 10^−4^ cm^2^ V^−1^  s^−1^), leading to balanced electron/hole (a ratio of 1.09) transport. Then, the relations between *J*_sc_ or *V*_oc_ and light intensity (*P*_light_) were further investigated to explore the different kinetics of charge recombination in diversified devices. In theoretical considerations, *V*_oc_ linearly depends on the light intensity with a slope of nk*T*/q (1 < *n* < 2), where k is Boltzmann’s constant, q is the elementary charge, n is a scaling factor, and *T* is Kelvin temperature. Trap-assisted recombination (or monomolecular recombination) is negligible with a slope of k*T*/q^52^. As seen in Supplementary Fig. 9a, *n* value decreases after HFSS, as well as TA and the film treated by PFMCH, shows the smallest value of 1.03, indicating that these post-treatments can suppress trap-assisted recombination. Supplementary Fig. 9b depicts the light intensity dependence of *J*_sc_. When α in the power-law dependence (*J*_sc_ ∝ *P*_light_^α^) is closer to unity, weaker bimolecular recombination will be achieved. Similarly, post-treatment would also benefit the mitigation of bimolecular recombination. Particularly, α value for the device with PFT treatment is the closest to unity (0.990). To deeply understand the charge recombination and extraction dynamics in varied devices, transient photovoltage (TPV) and transient photocurrent (TPC) were measured. From the voltage decay curves in Fig. 2e, the carrier lifetimes are evaluated to be 0.77 μs (pristine), 1.63 μs (TA), 1.78 μs (PFD), 1.93 μs (PFMCH), and 1.93 μs (PFT), respectively, suggesting these post-treatments in particular HFSS are able to surpass the charge recombination. As shown in TPC (Fig. 2f), the charge extraction time of pristine device under the short-circuit condition is 1.84 μs, which is as same as the TA treated device but obviously longer than those of devices treated by different fluorous solvents of PFD (1.70 μs), PFMCH (1.62 μs) and PFT (1.74 μs), demonstrating HFSS treated devices have higher charge extraction efficiencies. The TPV and TPC data are well consistent with the different FFs of OSCs, wherein BHJs treated by PFMCH and PFT show excellent FFs of 74.6 and 74.8%. These results clearly manifest HFSS can confidently outperform TA on enhancing charge mobility and restraining charge recombination.

### Microstructure and morphology

Based on the varied device parameters and photophysical behavior induced by different post-treatments, we could reason the molecular reorganization is performed at different levels, leading to the diversity of microstructure and morphology in BHJ. Firstly, the composite ratio of active layers is investigated by X-ray photoelectron spectroscopy (XPS). As shown in Supplementary Figs. 10 and 11, the signals of nitrogen element which only present in Y6 are almost identical among the blend films after post-treatments either with residue or without residue, indicating the change of PM6/Y6 ratio is negligible. Ultraviolet photoemission spectroscopy (UPS) reveals the diversity of surface energy levels among these blend films is very small (Supplementary Fig. 12), suggesting the improved performance is not mainly caused by the interfacial modification.

**Fig. 3:**
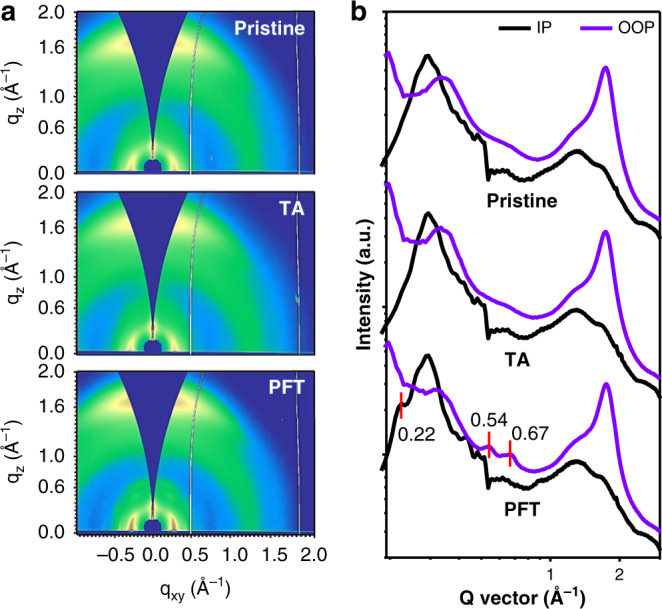
Microstructural characteristics. **a** GIWAXS 2D pattern and **b** corresponding 1D cutline profiles along the out-of-plane and in-plane directions of blend films prepared by different conditions.

Grazing-incidence wide-angle X-ray scattering (GIWAXS) is further characterized to investigate the microstructural difference. As shown in two-dimensional (2D) patterns (Fig. 3a and Supplementary Fig. 13a) and corresponding 1D plots along the out-of-plane (OOP) and in-plane (IP) directions (Fig. 3b and Supplementary Fig. 13b), these films with post-treatment present nearly identical features except the film with PFT treatment. Notably, three new diffraction peaks located at 0.22 Å^–1^ along IP and 0.54 Å^–1^ and 0.67 Å^–1^ along OOP are found in 1D plots of PFT-film. The first peak in the IP direction corresponding to a distance of 28.5 nm comes from Y6’s (020) diffraction and the third peak also originates from Y6 according to other reports^53^, indicating the existence of more ordered Y6 after PFT treatment. The much more ordered arrangement of Y6 after PFT treatment is consistent with the higher electron mobility aforementioned. The π–π stacking peaks of PM6 (*q*_z_ = 1.66 Å^–1^) and Y6 (*q*_z_ = 1.75 Å^–1^) are overlaid and could not be distinguished^23,42,53,54^. However, this peak area is obviously large in the film with PFT treatments. Besides, the crystalline size could be estimated by calculating the crystal coherence length (CCL) using the Scherrer equation. The CCL values of PFT based and pristine films are 20.3 Å and 19.4 Å in OOP direction, respectively. These results demonstrate molecular arrangement is much more ordered in PFT-treated film.

**Fig. 4:**
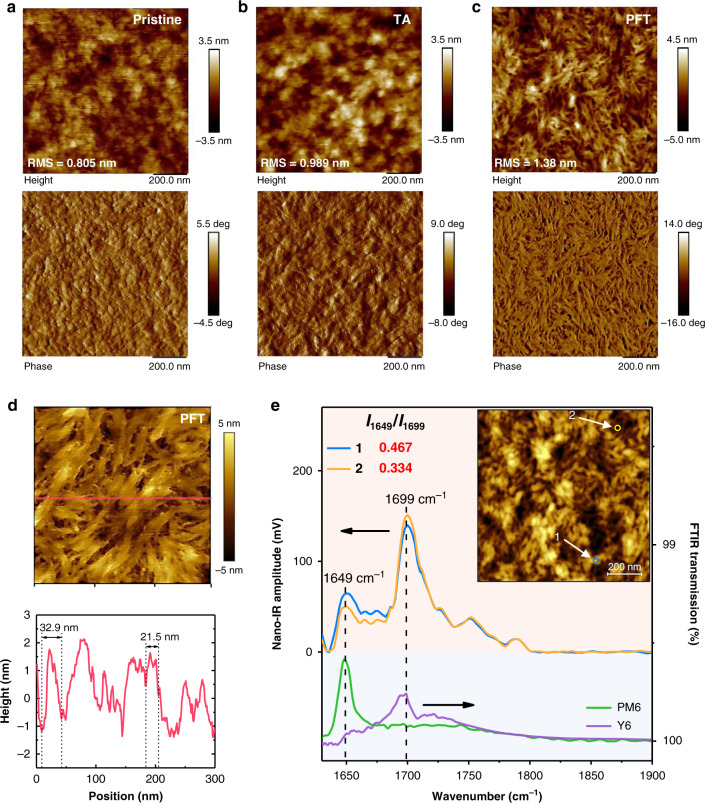
Morphology investigations. **a**–**c** AFM height (top) and phase (down) images of **a** pristine, **b** TA and **c** PFT-treated films. **d** AFM zoom-in image with high resolution (top) and height difference curve (down) of cutting line. **e** AFM topography image of PFT-treated films (top right) and corresponding Nano-IR spectra from the color-cycled positions (top), together with FTIR spectra of PM6 and Y6 (down).

To visualize the difference of morphology, these blend films are characterized by atomic force microscopy (AFM). As shown in Fig. 4a, the pristine blend film shows a uniform surface with the lowest root-mean-square surface roughness (RMS) of 0.805 nm. By elevating TA temperature to 90 °C, the RMS gradually increases to 0.989 nm (Fig. 4b and Supplementary Fig. 14), indicating the improved crystallinity as supported by larger grain size in both height and phase images. As we speculate, PFD acts as medium for thermal conduction, leading to the morphology of PFD-film resembles the one of 90 °C TA (Supplementary Fig. 15). Although PFMCH is processed at relatively low temperature (60 °C), the RMS significantly increased to 1.06 nm (Supplementary Fig. 15), which can be rationalized by that the mixed solvent containing residual CF/CN accelerates the molecular reorganization to increase the crystallinity. Dramatically, the film treated by PFT is profoundly transformed to well-defined fiber-like morphology with the biggest RMS of 1.38 nm (Fig. 4c), leading to significant phase separation as evidenced by phase image. To recognize the exquisite fiber structure, a zoom-in AFM height image with higher resolution is obtained as shown in Fig. 4d. These nanofibers appear as brush-like structure with sharp edge. The height difference of extracted lines (Fig. 4d and Supplementary Fig. 16) at slow-scan direction reveal semi-empirically that the domain size of fibers ranges from 10 to 50 nm, wherein most ones are 20–30 nm. To investigate the morphology of film interior, the blend film is etched by ion beam with different time^55^. As shown in Supplementary Fig. 17, fibrous network is also evident in freshly exposed surfaces which are localized at different positions in the vertical direction of blend film. The formation of fiber is also verified by TEM (Supplementary Fig. 18). TEM image verified the improved phase separation and the formation of fiber after PFT treatment, indicating the morphological optimization has already occurred not only on the surface but also in the interior of the blend film. The fiber-like network is beneficial for improving the efficiency of exciton dissociation due to the limited diffusion length of exciton (10–20 nm)^56–59^, while it also builds a suitable pathway to facilitate charge transport, and subsequently ensure excellent device performance.

To further explore the origin of fibrous phase in PFT based film, AFM-based nanometer-scaled infrared spectroscopy (nano-IR) has been employed, which combines the high spatial resolution of AFM with the chemical analysis ability of IR spectroscopy^60–63^. Since the stretching vibration of carbonyl groups of PM6 and Y6 exhibit distinct peaks at 1649 and 1699 cm^−1^ respectively in Fourier transform infrared spectrometer (FTIR, Supplementary Fig. 19), the nano-IR image of PFT-film at 1649 cm^−1^ (green) dividing that at 1699 cm^−1^ (purple) shows a clear contrast between PM6-rich domain and Y6-rich domain, and a continuous mesh of PM6 is evident (Supplementary Fig. 20). By taking nano-IR spectra at nanofiber stem (spot 1) and swale (spot 2) as shown in Fig. 4e, spot 1 shows higher intensity at 1649 cm^−1^ but lower intensity at 1699  cm^−1^ compared to spot 2. Comparing the ratios of peak intensity at 1649 cm^−1^ to that at 1699  cm^−1^, the larger ratio of spot 1 (0.467) than that of spot 2 (0.334) demonstrates that nanofibers are PM6 rich domains^60,61^. Based on these observations, it is clear that PFT treatment is able to transfer PM6 from grain to fiber-like structure in blend films, which explains improved hole mobility and more balanced charge transport in Fig. 2d. Combining the observation from GIWAXS and microscopies, it is clear the molecular reorganization induced by PFT treatment can enhance crystallinity of both PM6 and Y6, leading to improved phase separation in blend films.

### Universality of HFSS

The aforementioned results have clearly demonstrated that HFSS is able to significantly optimize the morphology of PM6:Y6 blend and then improve the photovoltaic performance of BHJ. To investigate the universality of HFSS, this method is also utilized in other donor/acceptor combinations (Supplementary Fig. 21). Considering the requirements of temperature and solubility, e.g., PFT should be processed below 100 °C due to its boiling point and PFT is not applicable with some donor/acceptor combinations containing small molecule due to its good solubility, the fluorous solvent was meticulously chosen in various donor/acceptor combinations. The device data are summarized in Supplementary Tables 8–14 and *J-V* curves are depicted in Supplementary Fig. 22. A brief summary of photovoltaic parameters of optimal devices are listed in Table 2. By replacing the acceptor with IT4F, TA seems to be less useful in PM6:IT4F system and the blend films without/with TA show similar RMS and 1.44 and 1.47 nm, respectively (Supplementary Fig. 23). However, the treatment of PFT (80 °C for 0.75 min) can visibly optimize the morphology with a RMS of 3.11 nm, leading to an increase of FFs from 74.7% (pristine) of 76.5% (PFT) and consequently providing a higher PCE of 13.10%. Another widely used donor/acceptor combination PTB7-Th:IEICO-4F was also studied. Due to the strong inter and intramolecular interactions of regioregular PTB7-Th^64,65^, TA (100 °C for 5 min) is difficult to advance the molecular reorganization, consequently resulting in similar PCEs of 11.22 and 11.29%, for pristine and TA treated devices respectively. When further increasing the temperature or elongating the processing time of TA, the efficiencies slightly decreased (Supplementary Table 9). Dramatically, an elevation of FFs from 66.6 to 71.3% is realized in the device with PFT treatment (70 °C for 0.5 min), providing a PCE of 12.23%. The AFM images of PTB7-Th:IEICO-4F blend film treated by PFT exhibit enhanced RMS and phase separation (Supplementary Fig. 24).

**Table 2:** The photovoltaic parameters of optimal devices based on various donor/acceptor combinations under the illumination of AM 1.5 G, 100 mW cm^−2^.

Active layer	Post-treatments	*V*_oc_ (V)	*J*_sc_ (mA cm^−2^)	FF (%)	PCE (%)
PM6:IT4F^a^	Pristine	0.86	19.93	74.7	12.81
	TA (100 °C for 5 min)	0.85	19.82	76.0	12.81
	PFT (80 °C for 0.75 min)	0.86	19.91	76.5	13.10
PTB7-Th:IEICO-4F^b^	Pristine	0.71	23.75	66.6	11.22
	TA (100 °C for 5 min)	0.70	23.84	67.7	11.29
	PFT (70 °C for 0.5 min)	0.71	24.15	71.3	12.23
P3HT:o-IDTBR^c^	Pristine	0.76	10.45	45.4	3.60
	TA (110 °C for 10 min)	0.75	13.24	60.0	5.96
	PFD (110 °C for 3 min)	0.75	13.25	61.9	6.15
P3HT:o-IDTBR^d^	Pristine	0.69	10.43	51.4	3.67
	TA (120 °C for 10 min)	0.74	12.11	67.5	6.05
	PFD (110 °C for 1 min)	0.73	12.61	71.3	6.56
BTR-Cl:Y6^e^	Pristine	0.93	4.52	22.8	0.96
	TA (120 °C for 5 min)	0.85	23.13	67.9	13.36
	PFD (120 °C for 2 min)	0.85	24.15	68.1	13.98
PTzBi-Si:N2200^f^	Pristine	0.89	14.21	56.9	7.20
	TA (120 °C for 5 min)	0.88	14.92	69.4	9.11
	PFD (120 °C for 1 min)	0.88	14.49	73.7	9.40

^a^CF:DIO (99.5:0.05, v/v) as processing solvent.

^b^CF:CN (96.5:3.5, v/v) as processing solvent.

^c^CF:CN (99.5:0.05, v/v) as processing solvent.

^d^Chlorobenzene as processing solvent.

^e^CF as processing solvent.

^f^2-Methyltetrahydrofuran as processing solvent.

Notably, the as-cast films of these three systems (PM6:Y6, IT4F:Y6 and PTB7-Th:IEICO-4F) already achieve relatively optimal morphology, which is demonstrated by the decent PCEs of the devices based on pristine blend films. To tap the potential of HFSS, this methodology is utilized in other donor/acceptor combinations whose pristine films have poor morphology. Among polymer donors, P3HT exhibits potential advantages for commercial applications due to its low-cost and facile manufacture, but the morphology optimization of devices based on P3HT is challenging and usually requires high-temperature and long-time TA. In P3HT:o-IDTBR blend films fabricated by chloroform solution with the additive of CN (99.5:0.5, v/v), the pristine device shows a poor PCE of 3.60% with a low FF of 45.4% indicating the unfavorable morphology. When the blend film is treated by TA at different temperature for 10 min, the increase of PCEs is very limited (Supplementary Table 10) until the temperature is exceeding 110 °C, wherein a PCE of 5.96% is achieved due to the improvement of FF (60.0%)^66^. When PFD is used, the tendency of PCE growth is similar with that of TA, since PFD is immiscible with CF/CN. Notably a higher PCE of 6.15% is realized in PFD case although the processing time (3 min) is much shorter than TA. On the contrary, HFSS treatment with PFMCH is able to significantly enhance the FFs and PCEs at relatively low temperature, for example, the devices treated by PFMCH and TA at 65 °C shows PCEs of 4.91 and 3.85% respectively (Supplementary Table 10). More importantly, a turning point, after which the rate of PCE growth dramatically increases, appears in the range of 50–60 °C (Supplementary Fig. 25). The behavior is identical with the case of PM6:Y6 wherein the same processing solvent is used, further verifying the miscibility of PFMCH with CF/CN above the upper critical solution temperature (around 50 °C) plays a key role in modulating the morphology of blend film and then enhancing the device performance. However, the low boiling point of PFMCH (76 °C) restrains the employment of high temperature (>100 °C). PFT with better miscibility was also tried, but o-IDTBR can partially dissolve in PFT even at room temperature (Supplementary Fig. 26a), limiting its application in P3HT:o-IDTBR. To further verify the thermal conduction and miscibility of fluorous solvent in P3HT:o-IDTBR system, the blend film was fabricated by chlorobenzene (CB) solution because the miscibility of PFD and CB gradually increases with elevating temperature (Supplementary Fig. 26b). As shown in Supplementary Fig. 27 and Supplementary Table 11, PFD can optimize the morphology at a shorter time (1 min), providing the champion PCE of 6.56%. When the residue of CB is removed by storing the as-cast film in vacuum for 12 h, the effectiveness of TA and PFD are similar (Supplementary Table 12), suggesting the CB residue is beneficial for the morphology modulation. These results demonstrate HFSS is available for blend films with either good or poor morphology, attributing to the synergistic effects of thermal conduction and miscibility of fluorous solvents.

Besides the combinations of polymer donor and small-molecule acceptor, all-polymer and all-small-molecule were also employed to verify the effectiveness of HFSS. In all-small-molecule system of BTR-Cl and Y6, the blend film spin-coated from chloroform solution exhibits poor photovoltaic performance with a very low PCE of 0.96%. With elevating the temperature of TA, the efficiencies are gradually enhanced^54^. Treating the blend film with PFD at 120 °C for 2 min can improve the photovoltaic performance with a PCE of 13.98% and a FF of 68.1%, which surpasses the best device with TA (a PCE of 13.36% and a FF of 67.9%). In PTzBi-Si:N2200 blends as a reported all-polymer OSCs^67^, the pristine device fabricated from 2-methyltetrahydrofuran (MeTHF) solution shows a moderate PCE of 7.20%. After TA at 120 °C for 5 min, the PCE is significantly increased to be 9.11% attributed to the enhancement of FFs from 56.9 to 69.4%. The FF can be further improved to 73.7% by PFD treatment (120 °C for 1 min), providing a PCE of 9.40%. Notably, all-small-molecule and all-polymer blend films are prepared with low boiling-point solvent CF or MeTHF, and then the solvent residue in fresh-made film is negligible, leading to the absence of mixture of solvent residue and fluorous solvent. Meanwhile, the fresh-made blend films (BTR-Cl:Y6 and PTzBi-Si:N2200) are stored in vacuum to ensure the solvent residue is completely removed before HFSS treatment, and consequently the devices shows similar efficiency with those without vacuum (Supplementary Table 15). Therefore, the improvement of device performance in these systems is mainly ascribed to the better thermal conductivity of fluorous solvent. Interestingly, the blend films with varied post-treatments show obviously different morphology. In PTzBi-Si:N2200 blend film, TA resulted in the co-existence of large fiber and tiny fiber with slight decline of RMS (from 1.19 to 1.07 nm), while PFD treatment leads to uniform fiber distribution with increased RMS of 1.28 nm (Supplementary Fig. 28) which is believed to be related with more uniform thermal conduction with HFSS.

In addition, the stability of devices with post-treatments was investigated. As shown in Supplementary Fig. 30, the device with PFT treatment shows similar decline tendency of TA-treated device, indicating that soaking in hot fluorous solvent would not bring extra defect for device stability.

## Discussion

In summary, by utilizing fluorous solvent with amphiphobic nature and temperature-dependent miscibility, a new post-treatment method namely HFSS is reported herein to optimize the morphology of BHJs. The improvement of device performance induced by HFSS surpasses the widely used TA in various BHJs. After the post-treatment of fluorous solvents, the photovoltaic performance in particular FF of PM6:Y6 system is significantly improved, providing a champion PCE of 16.52%, which surpasses the pristine device (15.21%) and the one (15.96%) with TA. Notably, this enhancement is accomplished on an efficient system of PM6:Y6, whereas the as-cast film has already achieved excellent morphology, suggesting HFSS is a robust method for morphological modulation. Systematical study unravels the versatility of fluorous solvent in the HFSS process. Firstly, as an orthogonal solvent with poor solubility to conjugated molecules, the fluorous solvent is applied as liquid medium to provide better thermal conduction, consequently accelerating the molecular reorganization. Moreover, the fluorous solvent penetrating into the blend film can be miscible with the residue of chloroform and chloronaphthalene to form a homogenous mixture above the upper critical solution temperature. This mixed solvent diffuses around the active layer, and then donor and acceptor molecules are selectively eroded and moved due to the tiny amount of CF/CN which has good solubility to organic semiconductor. With the synergic effects of heat and solvent, the molecular reorganization is performed quickly and completely in BHJ, leading to an ideal morphology with fiber-like PM6 and highly ordered Y6, which was confirmed by AFM, nano-IR, TEM and GIWAXS. Based on the morphology optimization, increased charge mobility and suppressed charge recombination are achieved in the optimal device, resulting in the enhancement of FF, eventually providing superior photovoltaic efficiency. More importantly, HFSS is applicable in various BHJs including polymer/small-molecule, all-polymer and all-small-molecule combinations, indicating the practicability and universality of this approach, in particular for large-scale manufacturing. In some cases like PM6:IT-4F and PTB7-Th:IEICO-4F, TA appears less useful while HFSS is still able to improve the photovoltaic performance. Meanwhile, compared to TA, HFSS is performed under relatively mild condition with lower temperatures and shorter processing time. Overall, this work builds a new avenue to take advantage of fluorous solvent with extraordinary amphiphobic nature and temperature-dependent miscibility on the application of morphological modulation. Besides OSCs, the film morphology is also vitally important in other fields of organic optoelectronic devices. Therefore, fruitful results are naturally expected by the wide application of HFSS in the near future.

## Methods

### Fabrication of OSC devices and HFSS treatment

Solar cell devices were fabricated in the configuration of the conventional sandwich architecture: indium tin oxide (ITO)/poly(3,4-ethylene dioxythiophene):polystyrene sulfonate (PEDOT:PSS)/PM6:Y6/ZnO/Ag. The patterned indium tin oxide glass (ITO) glass substrates (sheet resistance = 15 Ω sq^−1^) were cleaned in detergent, de-ion water, acetone, chloroform, acetone, and isopropanol sequentially by ultra-sonic bath for 15 min each and then dried by N_2_ gas. Further UV-Ozone treatment for 10 min was applied before use. The PEDOT-PSS solution was spin-coated onto the cleaned ITO glass substrate at 3000 rpm of 30 s followed by annealing at 150 °C of 15 min in the air to get about 35 nm thick. Then the PEDOT-PSS coated substrates were transferred into a nitrogen-filled glove box. The PM6:Y6 (1:1.2, weight ratio) were dissolved in chloroform (the concentration of blend solutions was 17.6 mg mL^−1^ in total), with the solvent additive of 1-chloronaphthalene (CN) (0.5%, v/v), and stirred at 55 °C for 2 h in a nitrogen-filled glove box. The blend solution was spin-coated at 3000 rpm for 30 s on the top of the PEDOT:PSS layer. For the device with TA, the spun-cast one was baked on a hot plate at various temperatures for 5 min. For the devices with HFSS treatment, the fresh-made spun-cast film was immersed into hot fluorous solvent with various temperatures and time followed by a drying step in vacuum for 0.5 h. The optimized thickness of the photoactive layer was about 120 nm measured by AFM. Then, a 10 mg/mL ZnO nanoparticle solution was spin-coated onto the active layer at 3000 rpm for 40 s. Finally, the anode, 100 nm Ag was deposited at a speed of 0.3 nm/s through a shadow mask by thermal evaporation in a vacuum chamber of under 2 × 10^−6^ Torr to complete the device fabrication. The active area of each device was defined to 3.64 mm^2^.

### AFM-based nanometer-scaled infrared spectroscopy

Nano-IR was obtained using a NanoIR3 (Anasys Instruments) coupled with pulse - tunable QCL source (Bruker; spectral resolution of 0.2 cm^−1^). An Au-coated silicon tapping mode NIR2 probe (PR-EX-TnIR-A-10, Bruker) was employed.

### Reporting summary

Further information on research design is available in the Nature Research Reporting Summary linked to this article.

## Supplementary information

Supplementary Information

Reporting Summary

## Data Availability

The data that support this paper and other findings of this study are available from the corresponding authors upon reasonable request.

## References

[1] Meng X (2019). A general approach for lab-to-manufacturing translation on flexible organic solar cells. Adv. Mater..

[2] Wang G, Adil MA, Zhang J, Wei Z (2019). Large-area organic solar cells: material requirements, modular designs, and printing methods. Adv. Mater..

[3] Liao C-Y (2020). Processing strategies for an organic photovoltaic module with over 10% efficiency. Joule.

[4] Ma X (2019). Achieving 14.11% efficiency of ternary polymer solar cells by simultaneously optimizing photon harvesting and exciton distribution. J. Mater. Chem. A.

[5] Schwarz KN (2020). Reduced recombination and capacitor-like charge buildup in an organic heterojunction. J. Am. Chem. Soc..

[6] Brus VV (2019). Solution-processed semitransparent organic photovoltaics: from molecular design to device performance. Adv. Mater..

[7] Shan T (2019). Achieving optimal bulk heterojunction in all-polymer solar cells by sequential processing with nonorthogonal solvents. ACS Appl. Mater. Interfaces.

[8] Zhao F, Wang C, Zhan X (2018). Morphology control in organic solar cells. Adv. Energy Mater..

[9] Lee H, Park C, Sin DH, Park JH, Cho K (2018). Recent advances in morphology optimization for organic photovoltaics. Adv. Mater..

[10] Kurpiers J (2018). Probing the pathways of free charge generation in organic bulk heterojunction solar cells. Nat. Commun..

[11] Kumari T, Lee SM, Kang S-H, Chen S, Yang C (2017). Ternary solar cells with a mixed face-on and edge-on orientation enable an unprecedented efficiency of 12.1%. Energy Environ. Sci..

[12] Kim Y, Yeom HR, Kim JY, Yang C (2013). High-efficiency polymer solar cells with a cost-effective quinoxaline polymer through nanoscale morphology control induced by practical processing additives. Energy Environ. Sci..

[13] Zhao J (2016). Efficient organic solar cells processed from hydrocarbon solvents. Nat. Energy.

[14] Ye L (2018). Quantitative relations between interaction parameter, miscibility and function in organic solar cells. Nat. Mater..

[15] He Y (2019). Evidencing excellent thermal-and photostability for single-component organic solar cells with inherently built-in microstructure. Adv. Energy Mater..

[16] Ghasemi M (2019). Delineation of thermodynamic and kinetic factors that control stability in non-fullerene organic solar cells. Joule.

[17] Du X (2019). Efficient polymer solar cells based on non-fullerene acceptors with potential device lifetime approaching 10 years. Joule.

[18] Ye L (2019). Quenching to the percolation threshold in organic solar cells. Joule.

[19] Chen Y, Zhan C, Yao J (2016). Understanding solvent manipulation of morphology in bulk-heterojunction organic solar cells. Chem. Asian J..

[20] Dong Y (2019). Ternary polymer solar cells facilitating improved efficiency and stability. Adv. Mater..

[21] Sun C (2019). Achieving fast charge separation and low nonradiative recombination loss by rational fluorination for high-efficiency polymer solar cells. Adv. Mater..

[22] Song J (2019). Ternary organic solar cells with efficiency >16.5% based on two compatible nonfullerene acceptors. Adv. Mater..

[23] Yuan J (2019). Single-junction organic solar cell with over 15% efficiency using fused-ring acceptor with electron-deficient core. Joule.

[24] Cui Y (2019). Over 16% efficiency organic photovoltaic cells enabled by a chlorinated acceptor with increased open-circuit voltages. Nat. Commun..

[25] An Q, Ma X, Gao J, Zhang F (2019). Solvent additive-free ternary polymer solar cells with 16.27% efficiency. Sci. Bull..

[26] Zhan L (2020). Over 17% efficiency ternary organic solar cells enabled by two non-fullerene acceptors working in an alloy-like model. Energy Environ. Sci..

[27] Fan B (2019). Achieving over 16% efficiency for single-junction organic solar cells. Sci. China-Chem..

[28] Yao Z (2018). Dithienopicenocarbazole-based acceptors for efficient organic solar cells with optoelectronic response over 1000 nm and an extremely low energy loss. J. Am. Chem. Soc..

[29] Sun H (2019). A monothiophene unit incorporating both fluoro and ester substitution enabling high-performance donor polymers for non-fullerene solar cells with 16.4% efficiency. Energy Environ. Sci..

[30] Ma R (2020). Improving open-circuit voltage by a chlorinated polymer donor endows binary organic solar cells efficiencies over 17%. Sci. China-Chem..

[31] Li S, Li C-Z, Shi M, Chen H (2020). New phase for organic solar cell research: emergence of Y-series electron acceptors and their perspectives. ACS Energy Lett..

[32] Huang W (2020). Efficient and mechanically robust ultraflexible organic solar cells based on mixed acceptors. Joule.

[33] Ye L (2013). Remove the residual additives toward enhanced efficiency with higher reproducibility in polymer solar cells. J. Phys. Chem. C.

[34] Yue Q (2019). 13.7% Efficiency small-molecule solar cells enabled by a combination of material and morphology optimization. Adv. Mater..

[35] Guo S, Cao B, Wang W, Moulin JF, Muller-Buschbaum P (2015). Effect of alcohol treatment on the performance of PTB7:PC71BM bulk heterojunction solar cells. ACS Appl. Mater. Interfaces.

[36] Xiao Z (2014). Universal formation of compositionally graded bulk heterojunction for efficiency enhancement in organic photovoltaics. Adv. Mater..

[37] Wang Y (2017). Engineering the vertical concentration distribution within the polymer: fullerene blends for high performance inverted polymer solar cells. J. Mater. Chem. A.

[38] Zhou W (2016). Surface treatment by binary solvents induces the crystallization of a small molecular donor for enhanced photovoltaic performance. Phys. Chem. Chem. Phys..

[39] Barthel-Rosa LP, Gladysz J (1999). Chemistry in fluorous media: a user’s guide to practical considerations in the application of fluorous catalysts and reagents. Coord. Chem. Rev..

[40] Matsubara, H. in *Encyclopedia of Physical Organic Chemistry* (ed. Wang, Z.) (Wiley & Sons, Inc., 2017).

[41] Zhang L (2020). Fabrication of collagen films with enhanced mechanical and enzymatic stability through thermal treatment in fluorous media. ACS Appl. Mater. Interfaces.

[42] Sun R (2020). A layer-by-layer architecture for printable organic solar cells overcoming the scaling lag of module efficiency. Joule.

[43] Perdigon-Toro L (2020). Barrierless free charge generation in the high-performance PM6:Y6 bulk heterojunction non-fullerene solar cell. Adv. Mater..

[44] Karki A (2019). Understanding the high performance of over 15% efficiency in single-junction bulk heterojunction organic solar cells. Adv. Mater..

[45] Tremolet de Villers BJ (2016). Removal of residual diiodooctane improves photostability of high-performance organic solar cell polymers. Chem. Mater..

[46] Zhao, W.-Z., Lu, B., Lv, S.-N., Zhou, C.-F. & Yang, Y. Simultaneous determination of chlorine and sulfur in geochemical reference samples by wavelength dispersive X-ray fluorescence spectrometry. *New J. Chem*. **44**, 11224–11230 (2020).

[47] Li X, Wang Y, Zhang Q (2015). Determination of halogen levels in marine geological samples. Spectrosc. Lett..

[48] Fontana MT (2018). Low-vapor-pressure solvent additives function as polymer swelling agents in bulk heterojunction organic photovoltaics. J. Phys. Chem. C..

[49] Yu R (2018). Design and application of volatilizable solid additives in non-fullerene organic solar cells. Nat. Commun..

[50] Bartesaghi D (2015). Competition between recombination and extraction of free charges determines the fill factor of organic solar cells. Nat. Commun..

[51] Lee TH (2016). Investigation of charge carrier behavior in high performance ternary blend polymer solar cells. Adv. Energy Mater..

[52] Proctor CM, Kuik M, Nguyen T-Q (2013). Charge carrier recombination in organic solar cells. Prog. Polym. Sci..

[53] Zhu L (2020). Efficient organic solar cell with 16.88% efficiency enabled by refined acceptor crystallization and morphology with improved charge transfer and transport properties. Adv. Energy Mater..

[54] Chen H (2019). All-small-molecule organic solar cells with an ordered liquid crystalline donor. Joule.

[55] Chen Q (2015). Quantitative operando visualization of the energy band depth profile in solar cells. Nat. Commun..

[56] Buchaca-Domingo E (2014). Additive-assisted supramolecular manipulation of polymer: fullerene blend phase morphologies and its influence on photophysical processes. Mater. Horiz..

[57] Shoaee S (2013). Charge photogeneration for a series of thiazolo-thiazole donor polymers blended with the fullerene electron acceptors PCBM and ICBA. Adv. Funct. Mater..

[58] Lee J (2019). Organic photovoltaics with multiple donor-acceptor pairs. Adv. Mater..

[59] Mikhnenko OV, Blom PWM, Nguyen T-Q (2015). Exciton diffusion in organic semiconductors. Energy Environ. Sci..

[60] Qiu B (2020). Highly efficient all-small-molecule organic solar cells with appropriate active layer morphology by side chain engineering of donor molecules and thermal annealing. Adv. Mater..

[61] Xue L (2017). Side chain engineering on medium bandgap copolymers to suppress triplet formation for high-efficiency polymer solar cells. Adv. Mater..

[62] Chen X, Lai J, Shen Y, Chen Q, Chen L (2018). Functional scanning force microscopy for energy nanodevices. Adv. Mater..

[63] Gu KL (2018). Nanoscale domain imaging of all-polymer organic solar cells by photo-induced force microscopy. ACS nano.

[64] Zhong H (2017). A regioregular conjugated polymer for high performance thick-film organic solar cells without processing additive. J. Mater. Chem. A.

[65] Zhong H, Li CZ, Carpenter J, Ade H, Jen AK (2015). Influence of regio- and chemoselectivity on the properties of fluoro-substituted thienothiophene and benzodithiophene copolymers. J. Am. Chem. Soc..

[66] Baran D (2017). Reducing the efficiency-stability-cost gap of organic photovoltaics with highly efficient and stable small molecule acceptor ternary solar cells. Nat. Mater..

[67] Li Z-Y (2019). Achieving efficient thick film all-polymer solar cells using a green solvent additive. Chin. J. Polym. Sci..

