# Supplementary material for: Universal and versatile morphology engineering via hot fluorous solvent soaking for organic bulk heterojunction
Source: Nat Commun. 2020 Nov 4;11:5585. doi: 10.1038/s41467-020-19429-x (PMC7642440; doi:10.1038/s41467-020-19429-x)
Supplement: Supplementary file 1 — Supplementary Information [file 41467_2020_19429_MOESM1_ESM.pdf]

SUPPLEMENTARY INFORMATION

for

Universal and Versatile Morphology Engineering via Hot Fluorous Solvent Soaking for Organic Bulk Heterojunction

Tong Shan, Yi Zhang, Yan Wang, Ziyi Xie, Qingyun Wei, Jinqiu Xu, Ming Zhang, Cheng Wang, Qinye Bao, Xin Wang, Chun-Chao Chen, Jingsong Huang, Qi Chen, Feng Liu, Liwei Chen and Hongliang Zhong

Content

1. Supplementary Methods
2. Additional Figures
3. Additional Tables

Supplementary Methods

General information. UV-vis absorption spectra were recorded on a SHIMADZU UV-2700 spectrophotometer. For the solid-state measurements, a suite of modules for holding up and fixing the ITO glass substrates was customized to ensuring the central area is measured. Atomic force microscopy (AFM) images were obtained using a Bruker Multimode 8 operated in tapping mode under air conditions at room temperature. High-resolution AFM image was performed using a Bruker ICON operated in ScanAsyst mode with a pixel density of 1024 × 1024. All thicknesses of films are detected by a Bruker Surface Profiler. GIWAXS characterization of the thin films was performed at the Advanced Light Source (Lawrence Berkeley National Laboratory) on beamline 7.3.3. Samples were prepared under device conditions on the Si/PEDOT:PSS substrates. The 10k eV X-ray beam was incident at a grazing angle of 0.16°. The crystal coherence length (CCL) was calculated using the Scherrer equation: $CL = 2\pi K/\Delta q$, where Δq is the full width at half-maximum of the peak, and K is a shape factor (0.94 was used here). XRF was performed by a sequential wavelength-dispersive X-ray fluorescence spectrometer (Shimadzu XRF-1800).

***Materials*.** The PTzBI-Si and N2200 were purchased from Dongguan Alda New Materials Technology Co., Ltd. Other organic materials were synthesized in lab. Perfluorodecalin (PFD) was purchased from Sigma-Aldrich. Perfluoro(methylcyclohexane) (PFMCH) was purchased from J&K Chemicals. Perfluorotoluene (PFT) was purchased from Shangfluoro. Methanol was purchased from Acros. Chloroform (CF) was purchased from Adamas-beta. 1-chloronaphthalene (CN) was purchased from TCI. Zinc acetate and potassium hydroxide were purchased from Alfa.

Device characterization. The device J-V characteristics were recorded by a Keithley 2420 SourceMeter unit in forward direction under AM 1.5G 1 sun irradiance (100 mW cm^{-2}) as generated by a 300 W Xe lamp solar simulator (Enlitech SS-F5-3A) at room temperature. The light intensity were calibrated using a standard Si diode with KG-5

filter. The EQE spectra were characterized using an Enlitech EQE system (Enlitech QE-M110) with a Si diode as reference cell. Monochromatic light was generated from an Enlitec lamp source with a monochromator. Transient photovoltage (TPV) and transient photocurrent (TPC) measurements were performed with Paios system (FLUXiM AG, Switzerland).

Charge carrier mobility measurement. Hole-only diode configuration: ITO/PEDOT-PSS/active layers/MoO_3/Al. Electron-only diode configuration: ITO/ZnO/active layers/ZnO/Al. PEDOT:PSS, MoO_3, ZnO, and Al were deposited by the same route as OSC devices.

The mobility in active layers were determined by fitting the dark current hole/electron-only diodes to the space-charge limited current (SCLC) model. The mobility was determined by the equation (1):

$$J = \frac{9\varepsilon_0\varepsilon_r\mu_0 V^2}{8L^3} \qquad (1)$$

where *J* is current density, μ is the hole or electron mobility, ε_0 is the dielectric permittivity of the active layer (generally assumed to be 3 for organic materials), ε_0 is the dielectric permittivity of free space (ε_0 = 8.854187817 × 10^{-12} F/m), L is the film thickness, and *V* is the voltage, which is defined as $V = V_{appl} - V_{bi}$, where V_{appl} is the applied voltage, V_{bi} is the built-in voltage.

Additional Figures

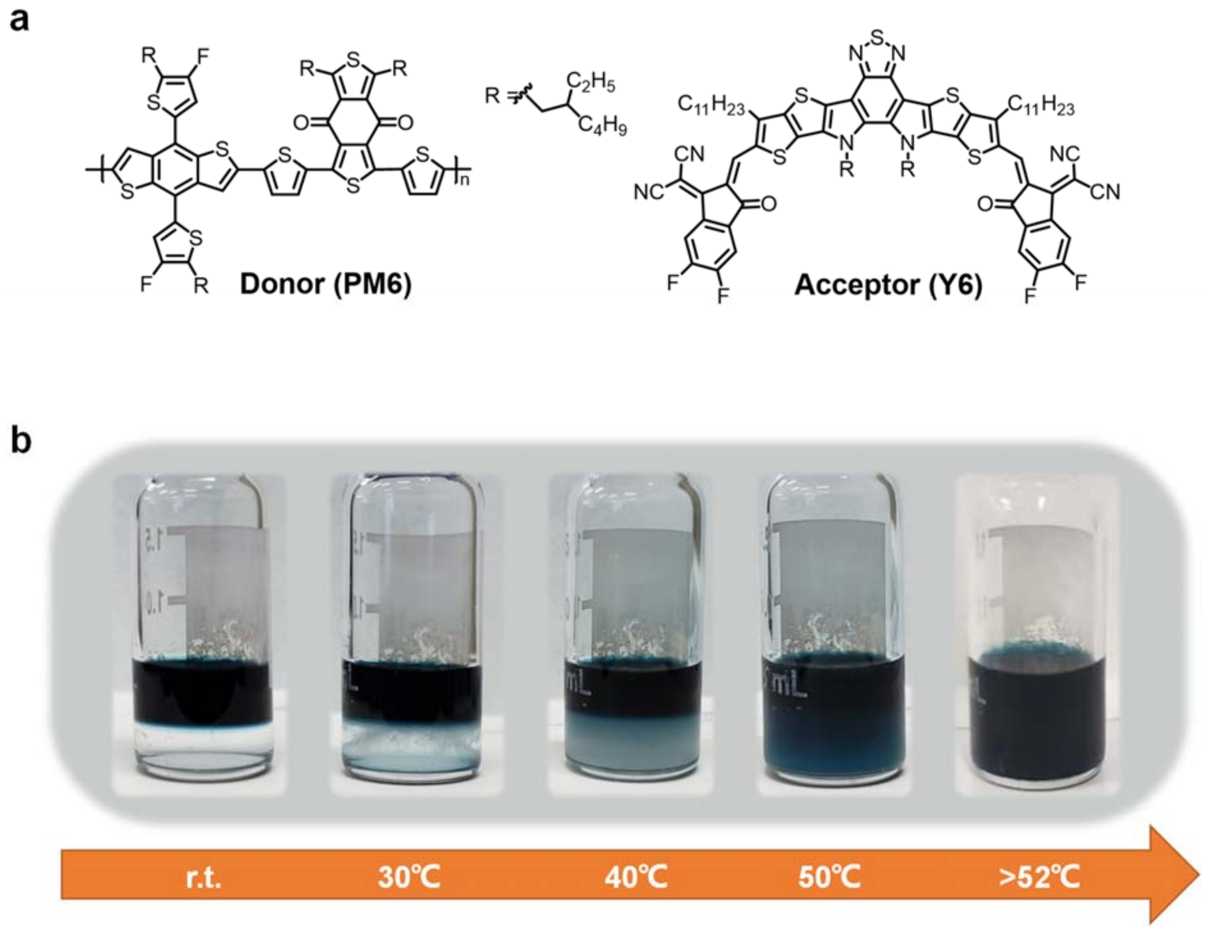

Supplementary Fig. 1 a Chemical structures and temperature-dependent miscibility fluorous solvents. Chemical structures of the donor and acceptor materials investigated in this work. **b** Photos of temperature-dependent miscibility of PFMCH and chloroform/chloronaphthalene (v/v, 1:1:0.05) (Y6 as a color agent dissolved in chloroform).

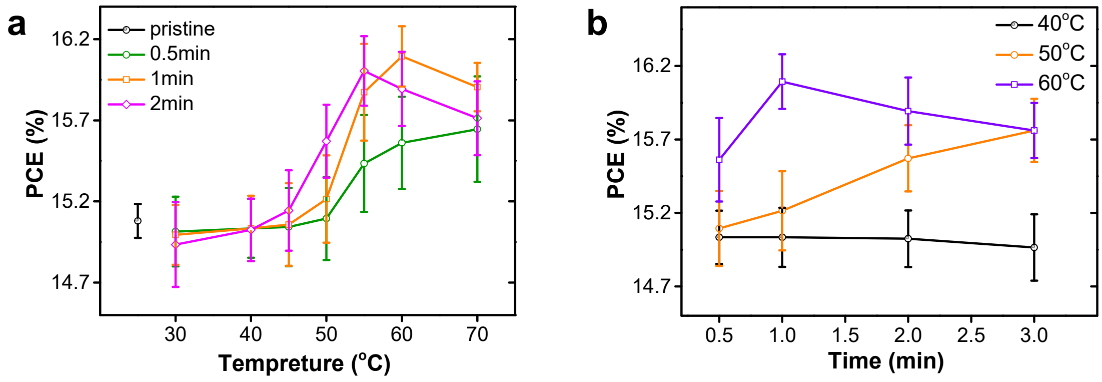

Supplementary Fig. 2 Photovoltaic parameters of PFMCH-treated devices. a and **b** PCEs change of PFMCH-treated PM6:Y6 devices with different time and temperatures. Error bars represent one standard deviation from the mean (n = 12).

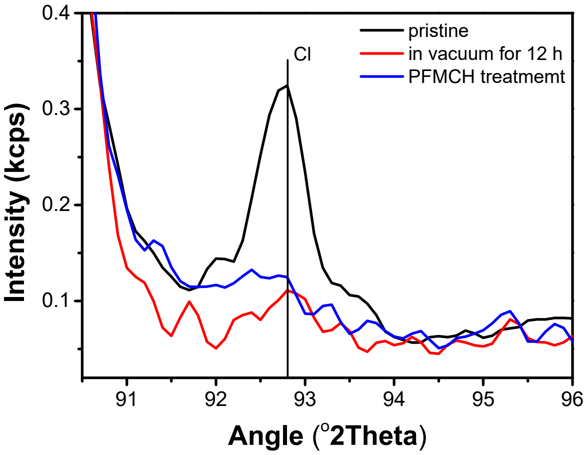

Supplementary Fig. 3 XRF spectra of PM6:Y6 fresh-made pristine film, with storage in vacuum for 12 hours, and with PFMCH treatment at 60 °C for 1 min in the Cl Ka wavelength range, Rh-tube, 40 kV/95 mA, and Ge111.

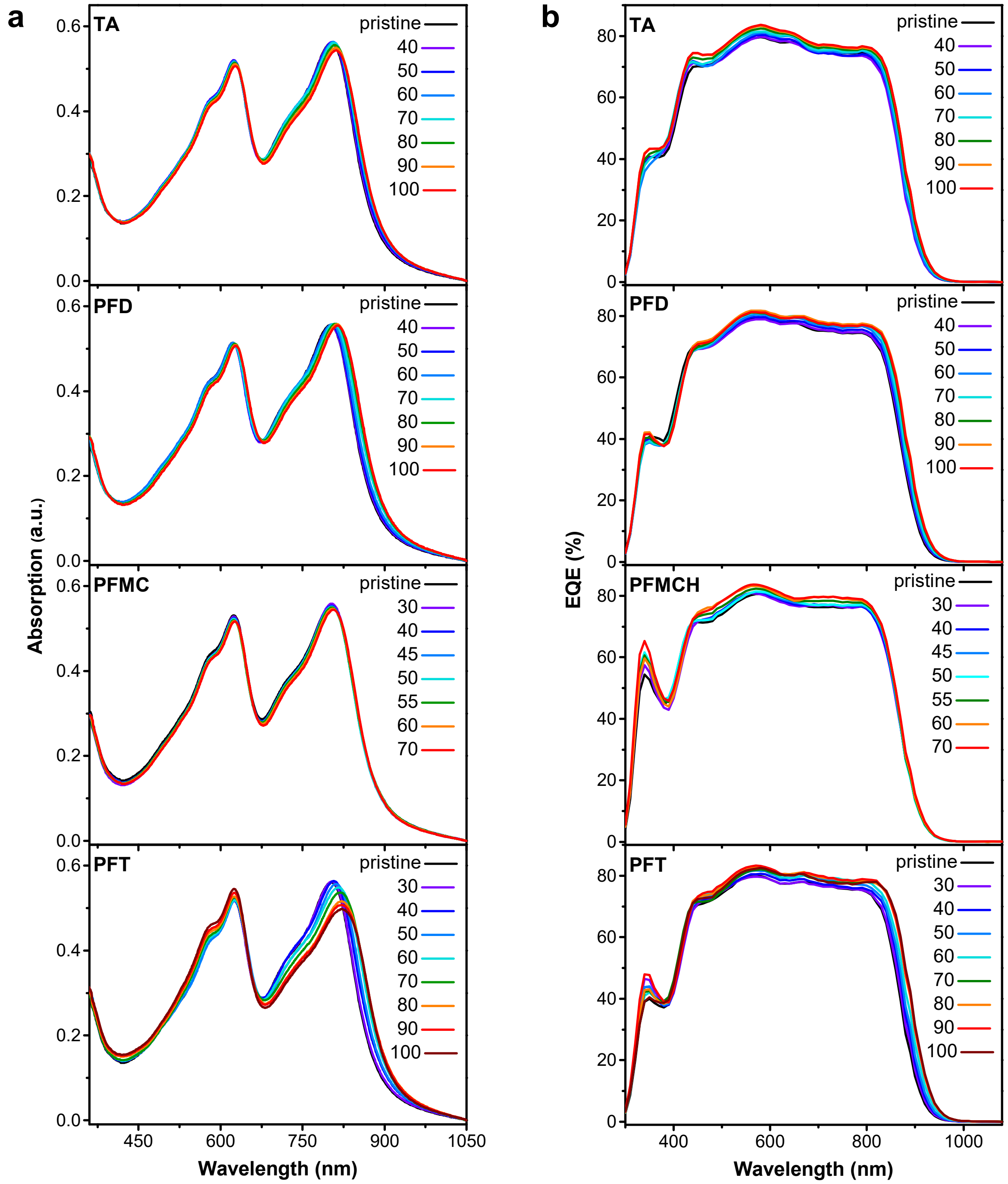

Supplementary Fig. 4 Absorption and corresponding EQE spectra change. a Absorption spectra of PM6:Y6 active layers with different post-treatments at various temperatures. **b** EQE spectra of devices with different post-treatments at various temperatures.

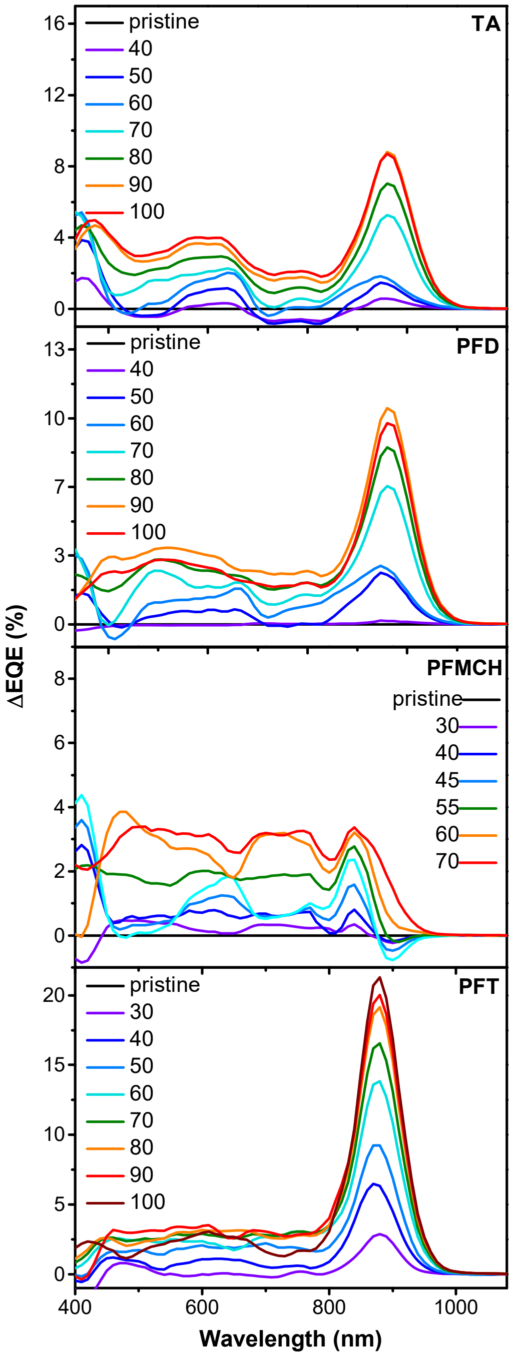

Supplementary Fig. 5 The change of EQE spectra of PM6:Y6 devices with different post-treatments at various temperatures.

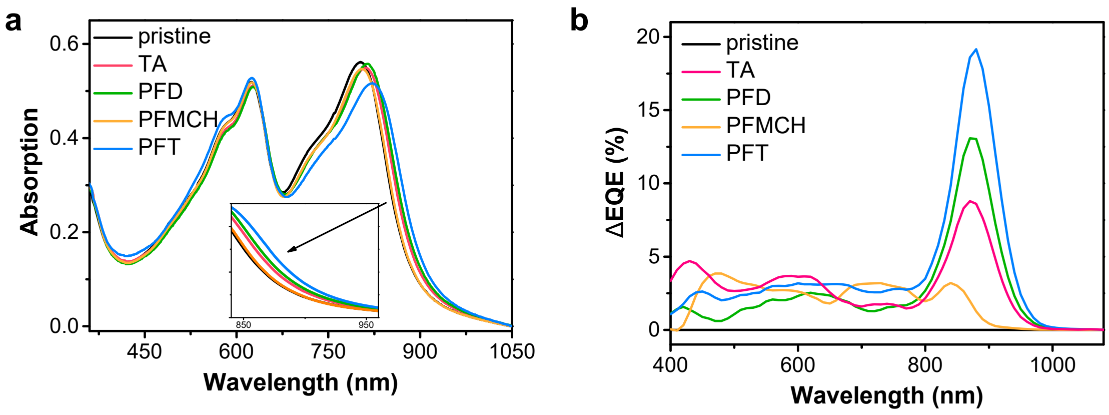

Supplementary Fig. 6 Absorption spectra and the change of EQE spectra of PM6:Y6 active layers with different post-treatments at optimized condition. a Absorption spectra. **b** The change of EQE spectra.

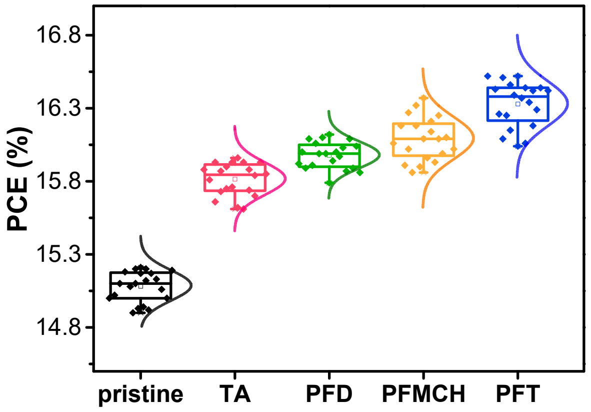

Supplementary Fig. 7 PCE of optimized PM6:Y6 devices depicted as standard box plots. Parameter spatial statistics (min, first quartile, median, mean, third quartile, and max; n = 20).

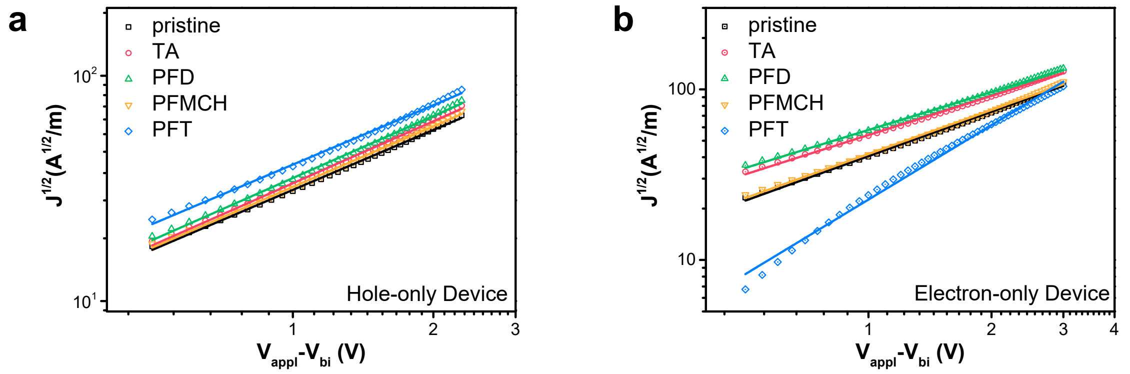

Supplementary Fig. 8 Fitting *J-V* curves of PM6:Y6 single carrier diodes with different post-treatments in SCLC model. a hole-only devices. **b** electron-only devices.

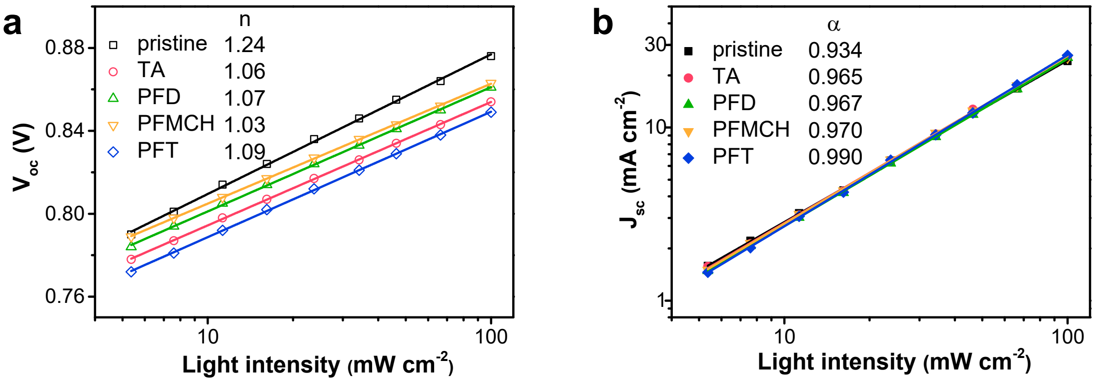

Supplementary Fig. 9 Charge recombination characterization. a V_{oc} and **b** J_{sc} versus light intensity of PM6:Y6 devices with different post-treatments at optimized condition.

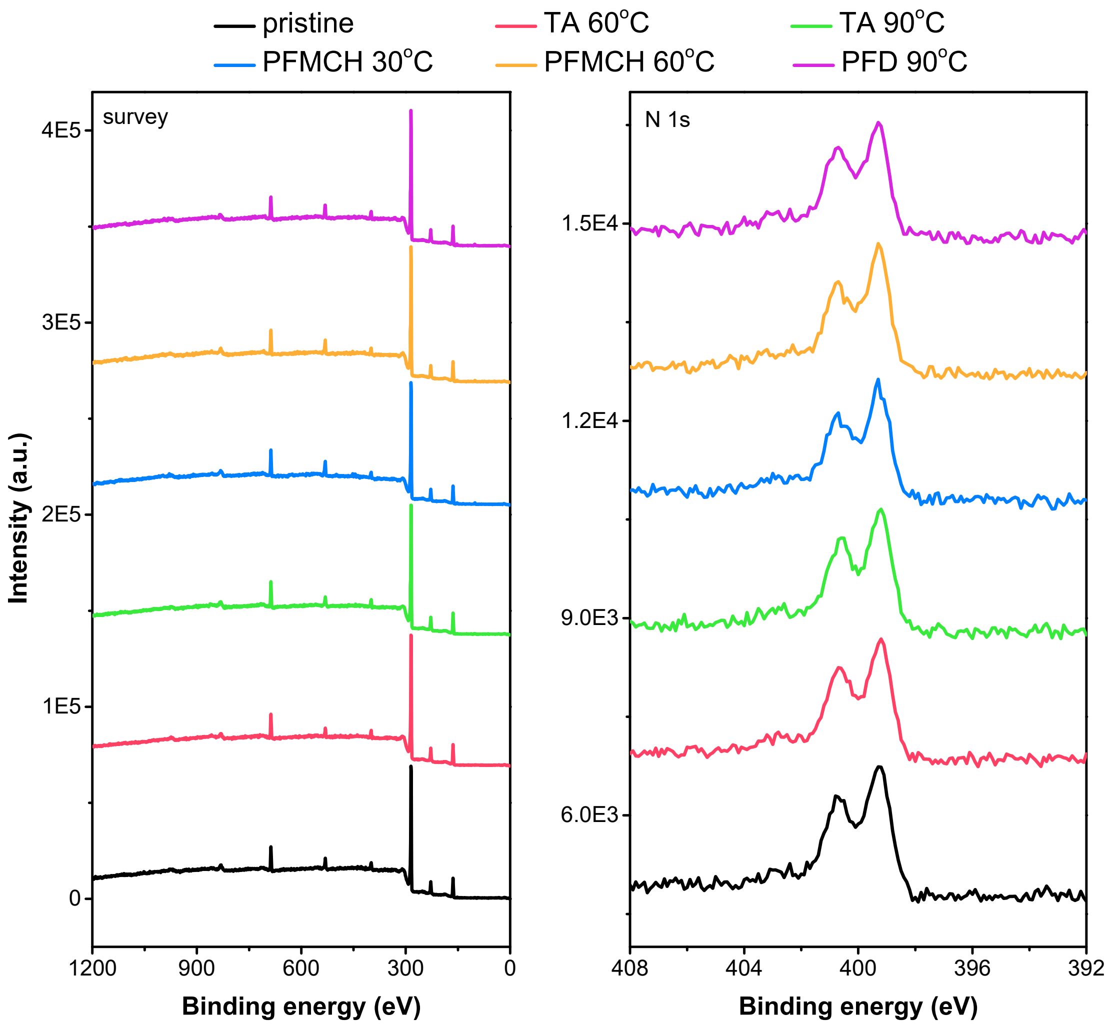

Supplementary Fig. 10 Survey XPS spectra and fine scan spectra focusing on the N1s peak for glass/ITO/PEDOT-PSS/PM6:Y6 active layer (post-treatments with processing solvents residues) samples.

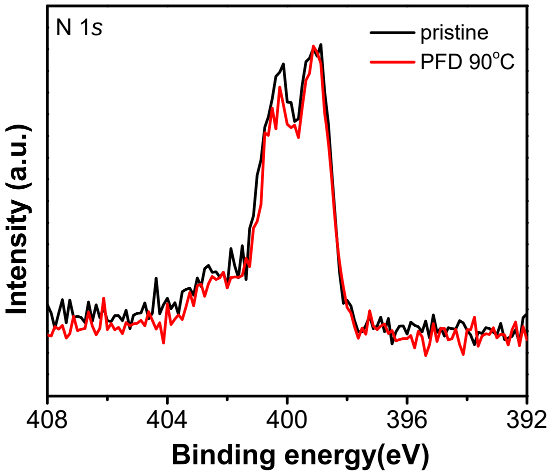

Supplementary Fig. 11 Fine scan XPS spectra focusing on the N 1*s* peak for glass/ITO/PEDOT-PSS/PM6:Y6 active layer (post-treatment without processing solvents residues) samples.

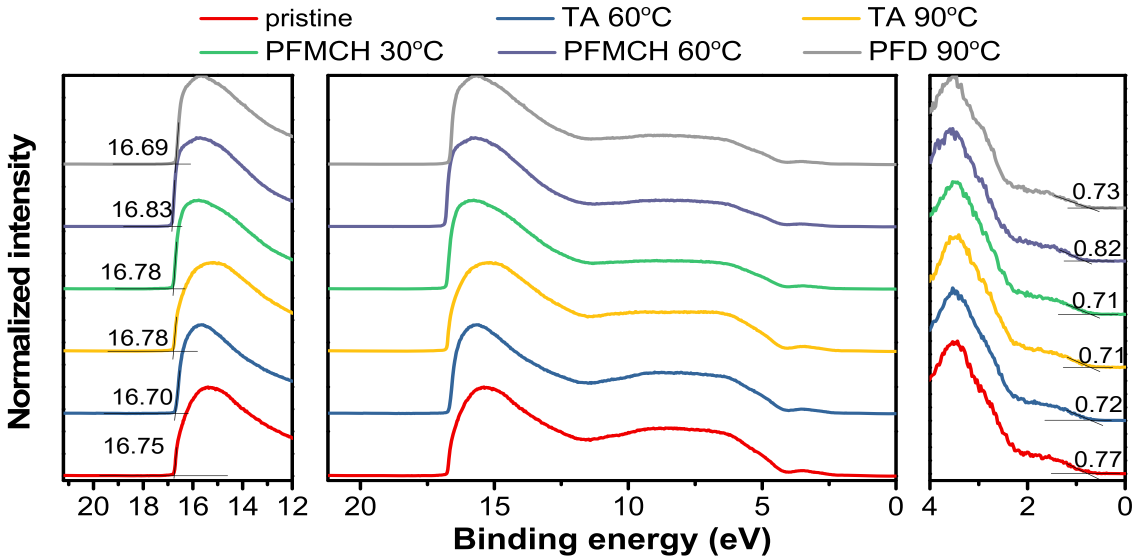

Supplementary Fig. 12 UPS spectra for glass/ITO/PEDOT-PSS/PM6:Y6 active layer (post-treatments with processing solvents residues) samples.

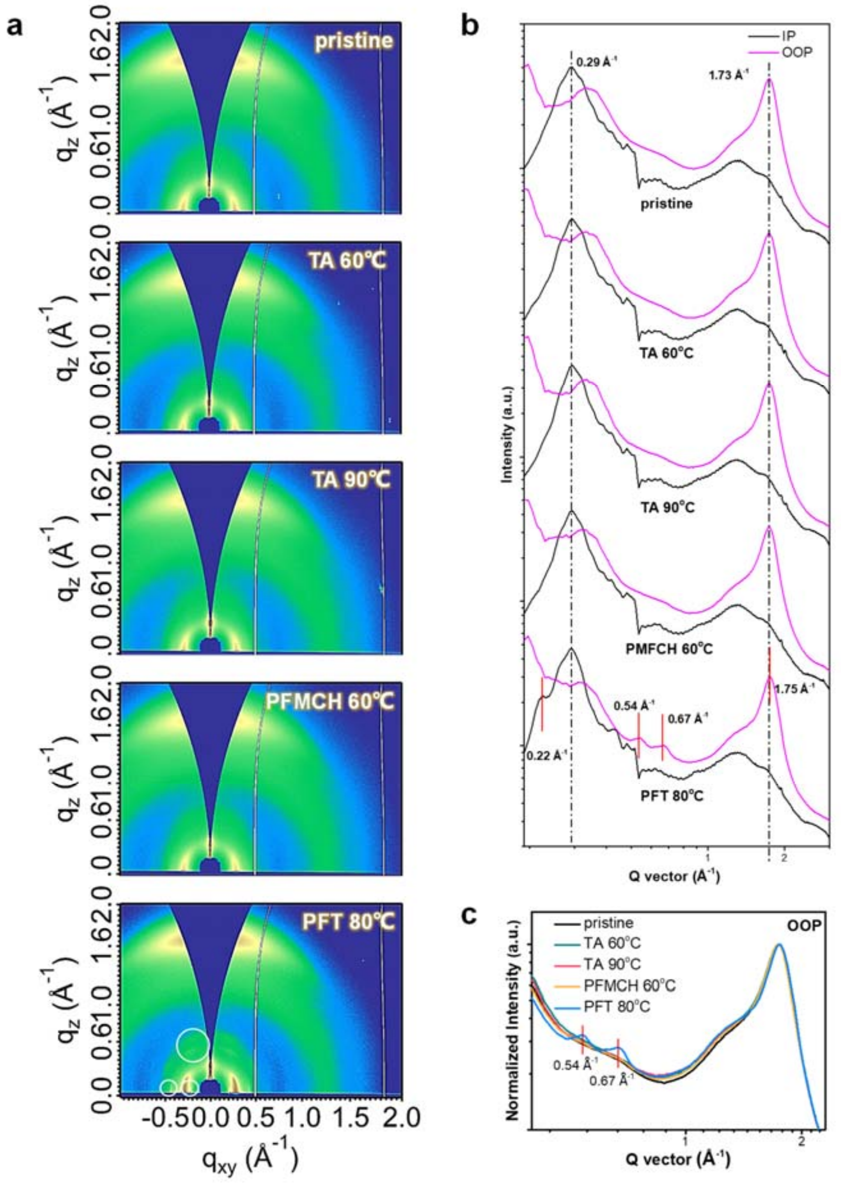

Supplementary Fig. 13 Microstructural characteristics. a GIWAXS 2D patterns of PM6:Y6 active layers with different post-treatments. **b** GIWAXS 1D cutline profiles extracted from 2D patterns along the out-of-plane and in-plane directions. **c** Normalized GIWAXS 1D cutline profiles along the out-of-plane direction.

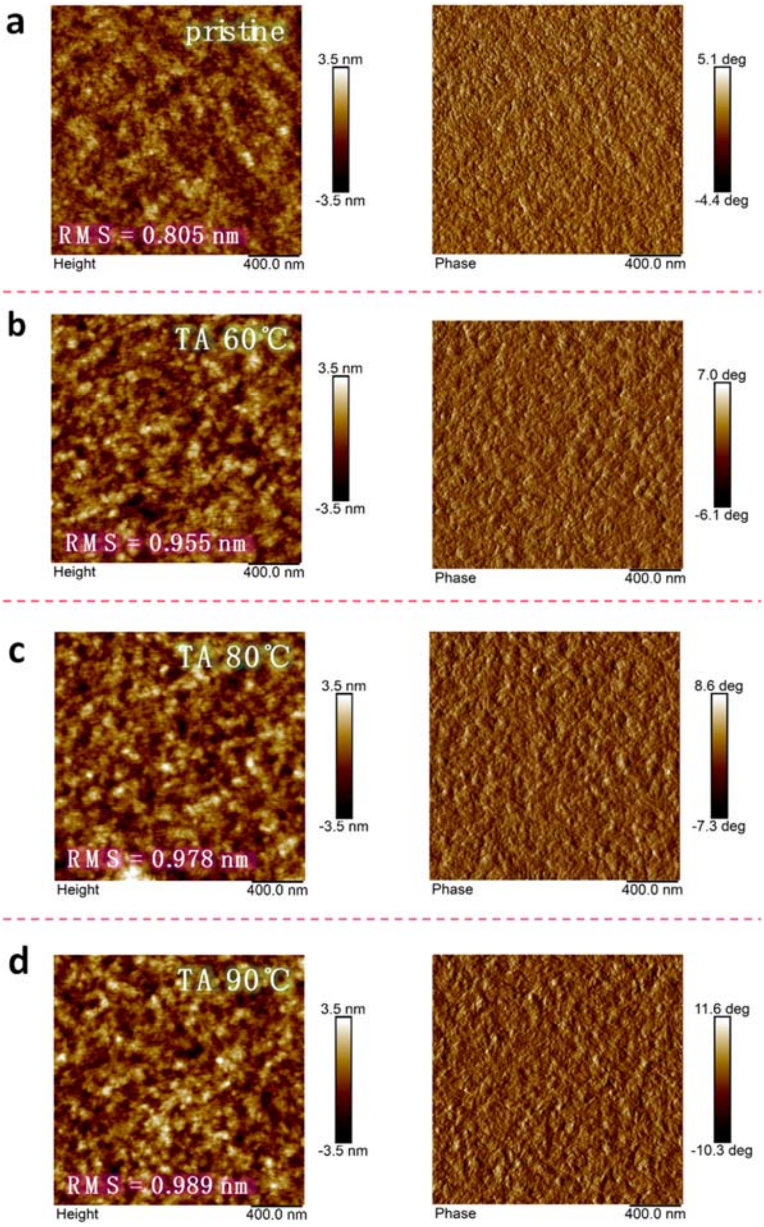

Supplementary Fig. 14 Morphology of PM6:Y6 active layers with TA at different temperatures. a to **d** AFM height images and phase images.

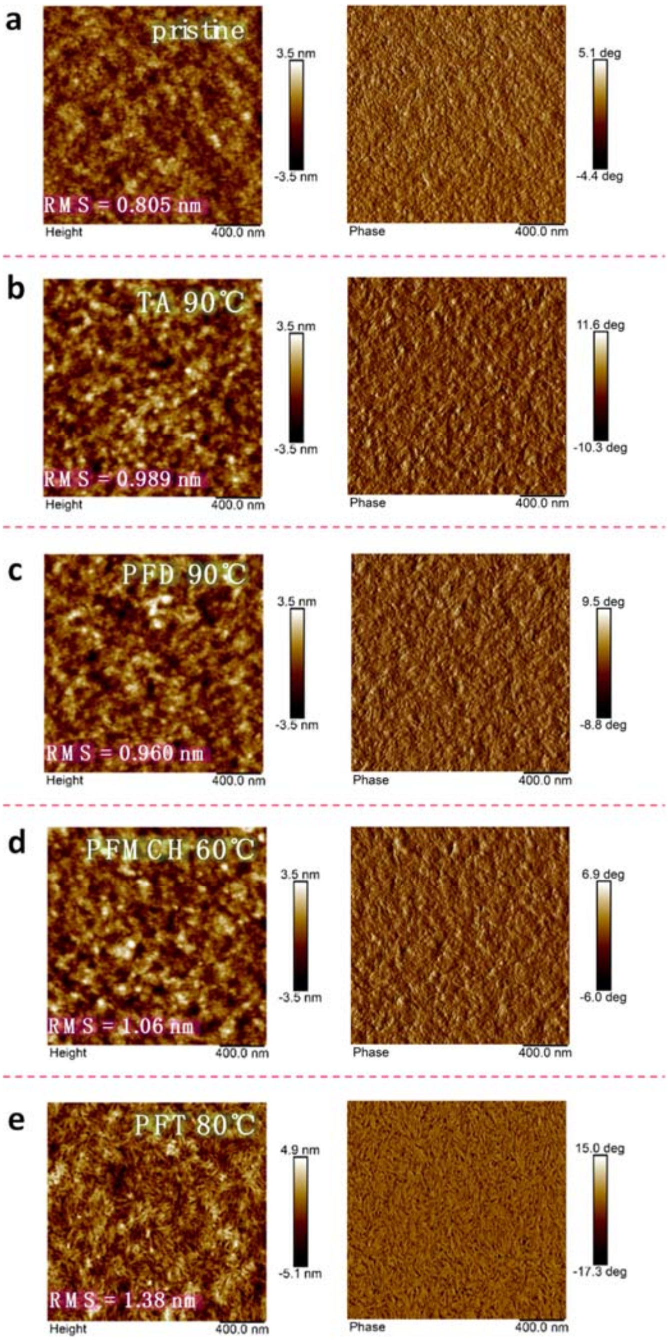

Supplementary Fig. 15 Morphology of PM6:Y6 active layers with TA at different post-treatments. a to **e** AFM height images and phase images.

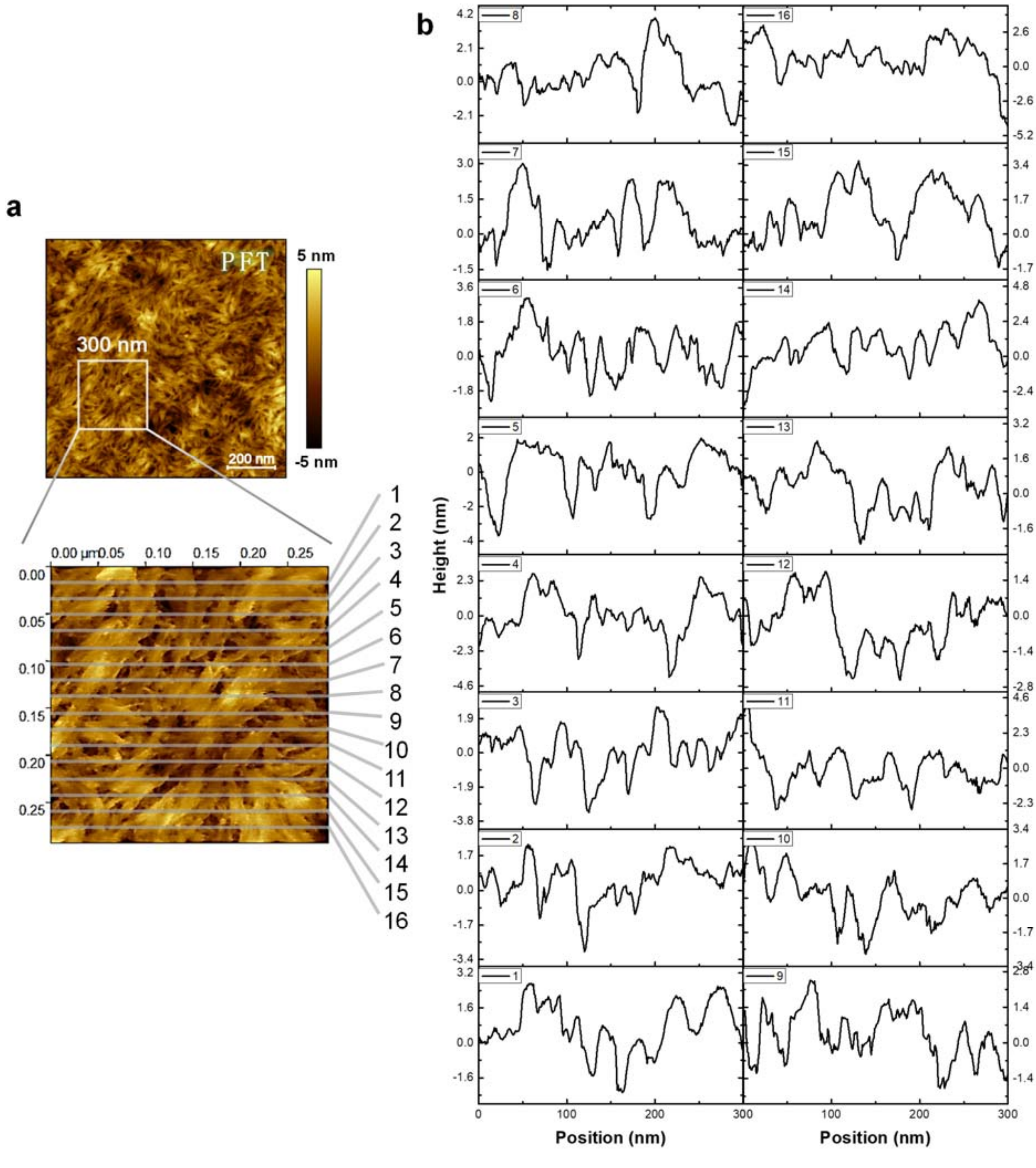

Supplementary Fig. 16 Fine structures of PFT-treated PM6:Y6 film with high resolution. a AFM images (top) and partial enlarged region (down). **b** Height difference curves of cutting lines (labeled by different numbers) from partial enlarged region.

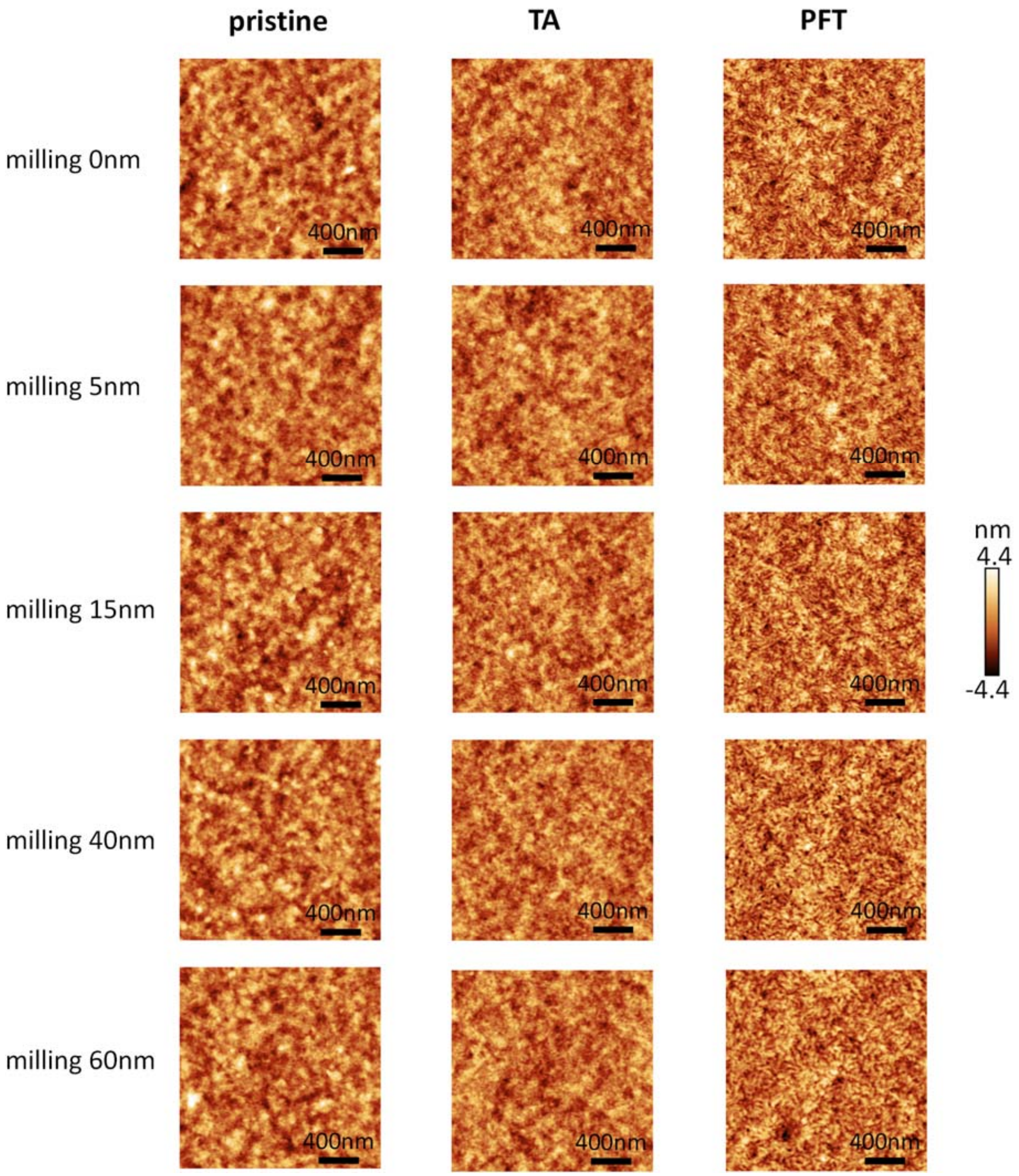

Supplementary Fig. 17 The topography change upon ion beam etching in pristine, TA-treated, and PFT-treated PM6:Y6 films. The thickness of the film after etching was very coarsely controlled by the etching time.

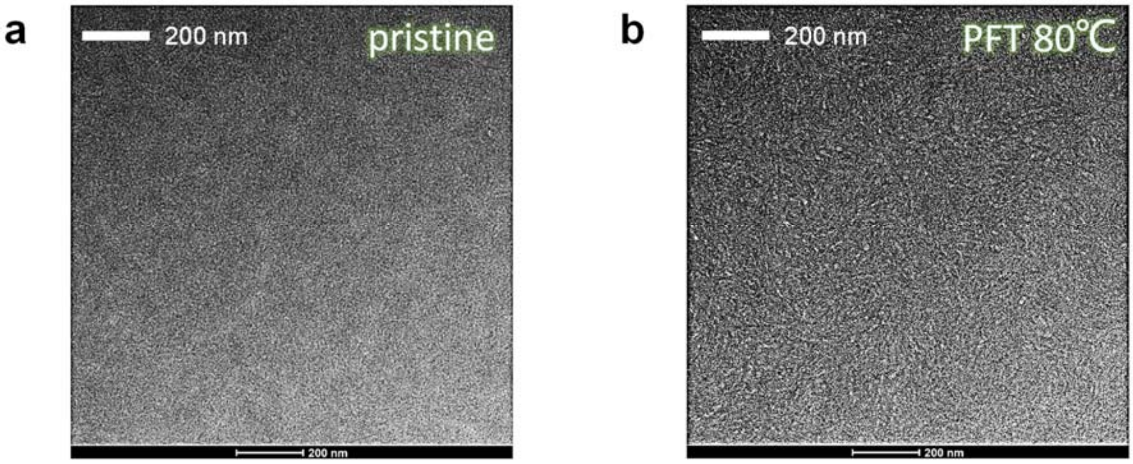

Supplementary Fig. 18 TEM images of PM6:Y6 film. a pristine film. **b** PFT-treated film.

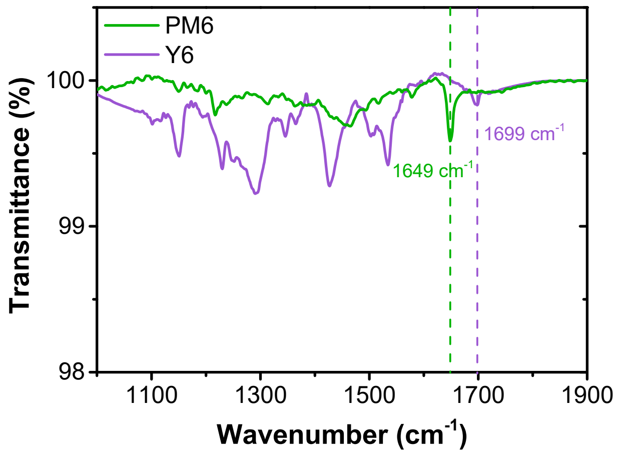

Supplementary Fig. 19 FTIR spectra of PM6 and Y6. The characteristic peak at 1649 cm^{-1} was selected for PM6, and the characteristic peaks at 1699 cm^{-1} was selected for Y6.

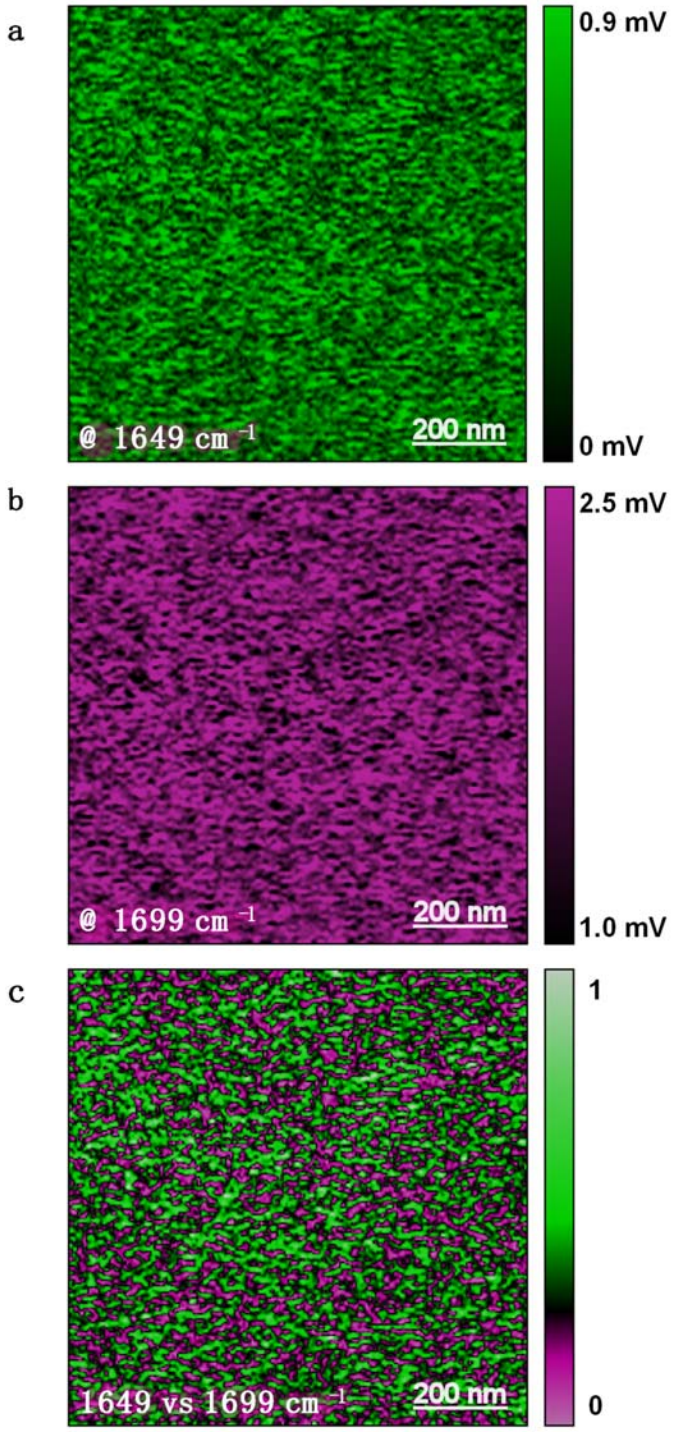

Supplementary Fig. 20 AFM-based nanometer-scaled infrared spectroscopy (nano-IR). a and **b** IR mapping image of PFT-treated films mapped at 1649 cm^{-1} (PM6) and 1699 cm^{-1} (Y6). **c** IR mapping image based on dividing the signal at 1649 cm^{-1} by that at 1699 cm^{-1}.

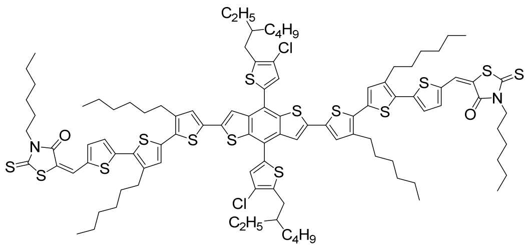

BTR-Cl

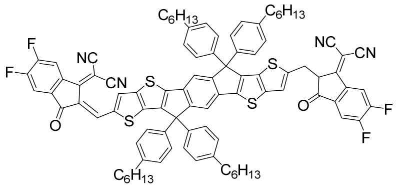

IT4F

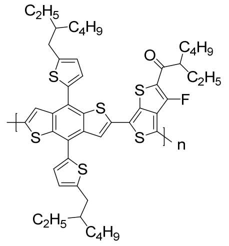

PTB7-Th

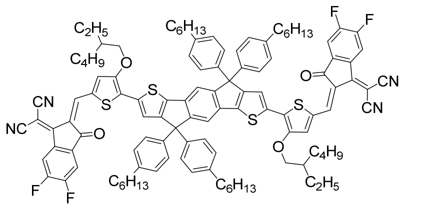

IEICO-4F

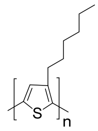

P3HT

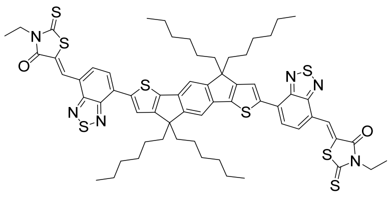

o-IDTBR

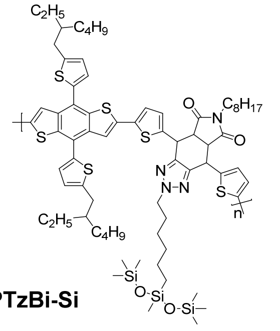

PTzBi-Si

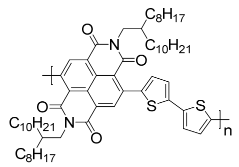

N2200

Supplementary Fig. 21 Chemical structures of other donor and acceptor materials for the investigation of universality of HFSS in this work.

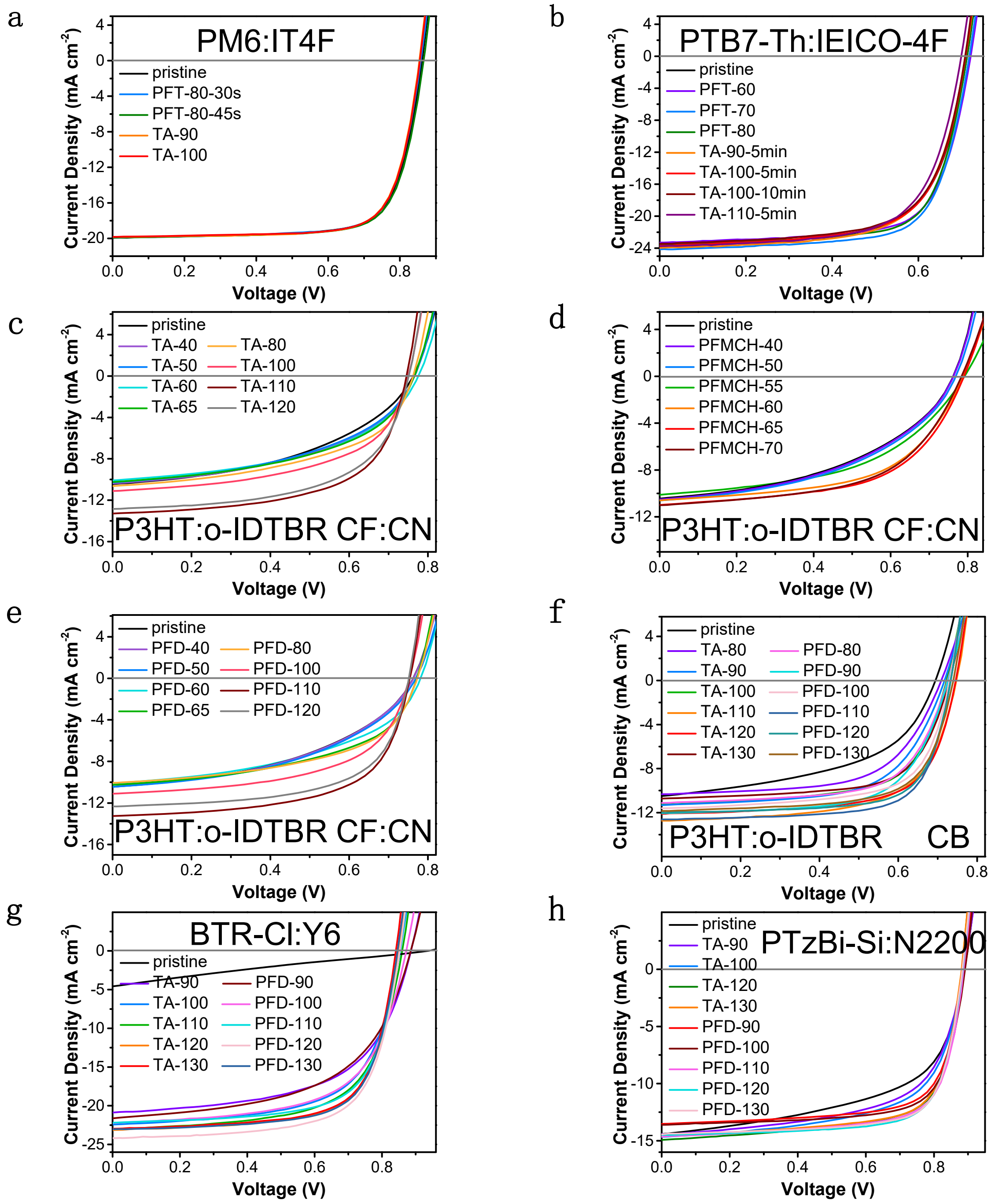

Supplementary Fig. 22 *J*−*V* curves of devices based on other active layer systems with various post-treatments. a PM6:IT4F. **b** PTB7-Th:IEICO-4F. **c to f** P3HT:o-IDTBR. **g** BTR-Cl:Y6. **h** PTzBi-Si:N2200.

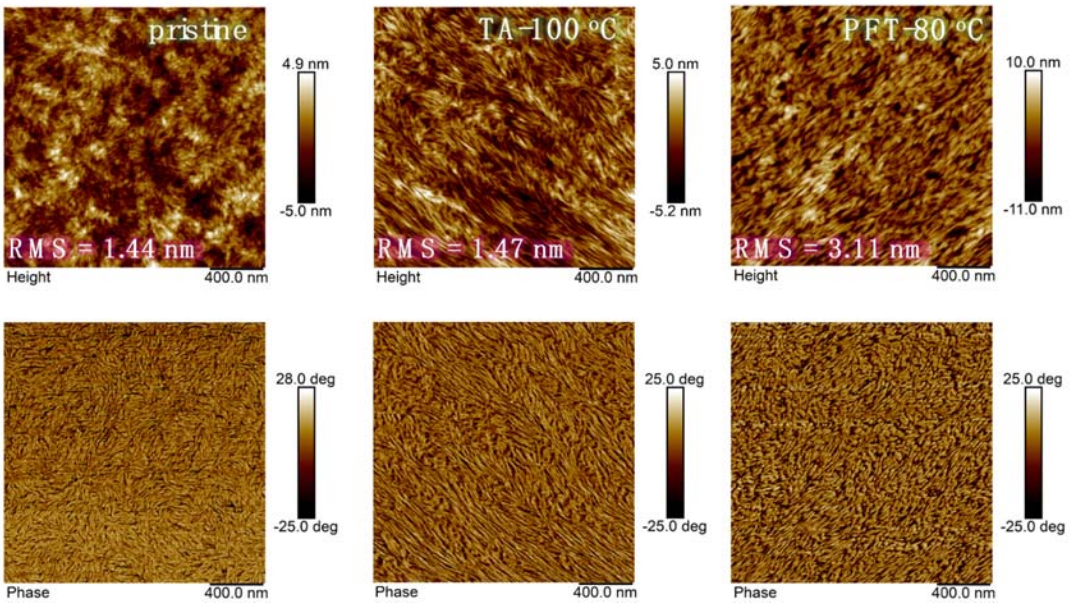

Supplementary Fig. 23 AFM height images and phase images of PM6:IT4F films on the condition of pristine, TA-treated at 100 °C for 5min, and PFT-treated at 80 °C for 0.75 min, respectively.

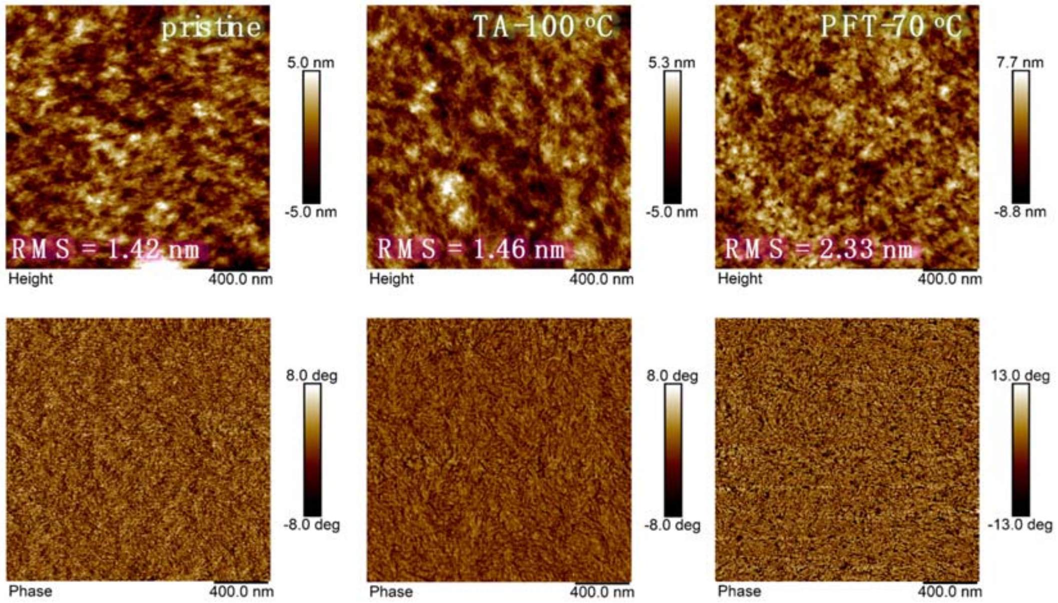

Supplementary Fig. 24 AFM height images and phase images of PTB7-Th:IEICO-4F films on the condition of pristine, TA-treated at 100 °C for 5min, and PFT-treated at 70 °C for 0.5 min, respectively.

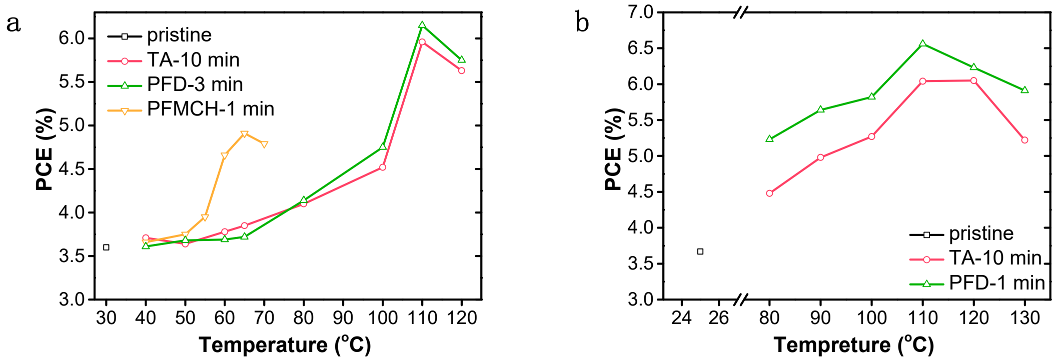

Supplementary Fig. 25 PCE versus temperature of post-treatments of P3HT:o-IDTBR devices processed by different solvents. a CF:CN (99.5:0.5, v/v) as processing solvent. **b** CB as processing solvent.

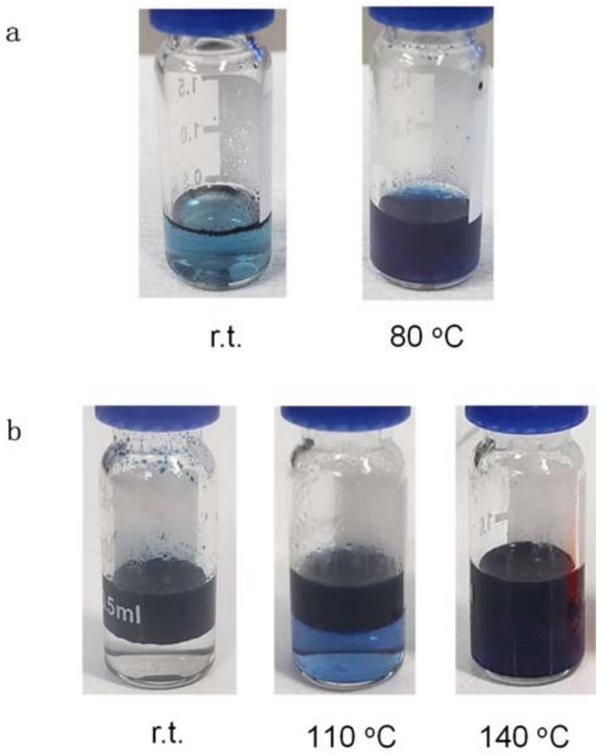

Supplementary Fig. 26 Solubility of o-IDTBR in PFT and miscibility of PFD and CB. a Photos of o-IDTBR in PFT at different temperatures. **b** Photos of temperature-dependent miscibility of PFD and CB (v/v, 1:1) (o-IDTBR as a color agent).

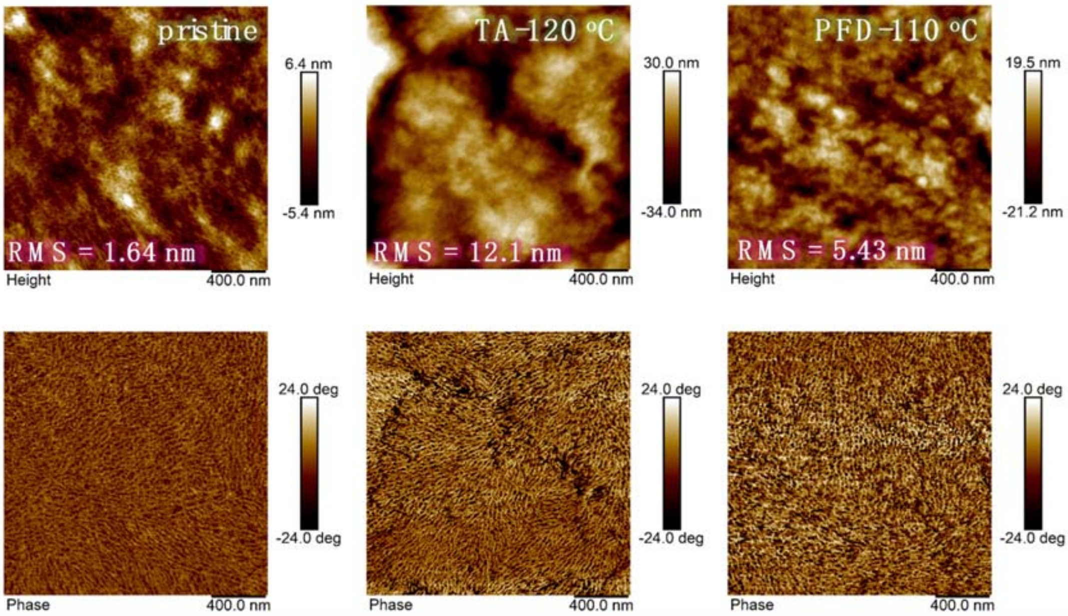

Supplementary Fig. 27 AFM height images and phase images of P3HT:o-IDTBR films on the condition of pristine, TA-treated at 120 °C for 10min, and PFD-treated at 110 °C for 1 min, respectively.

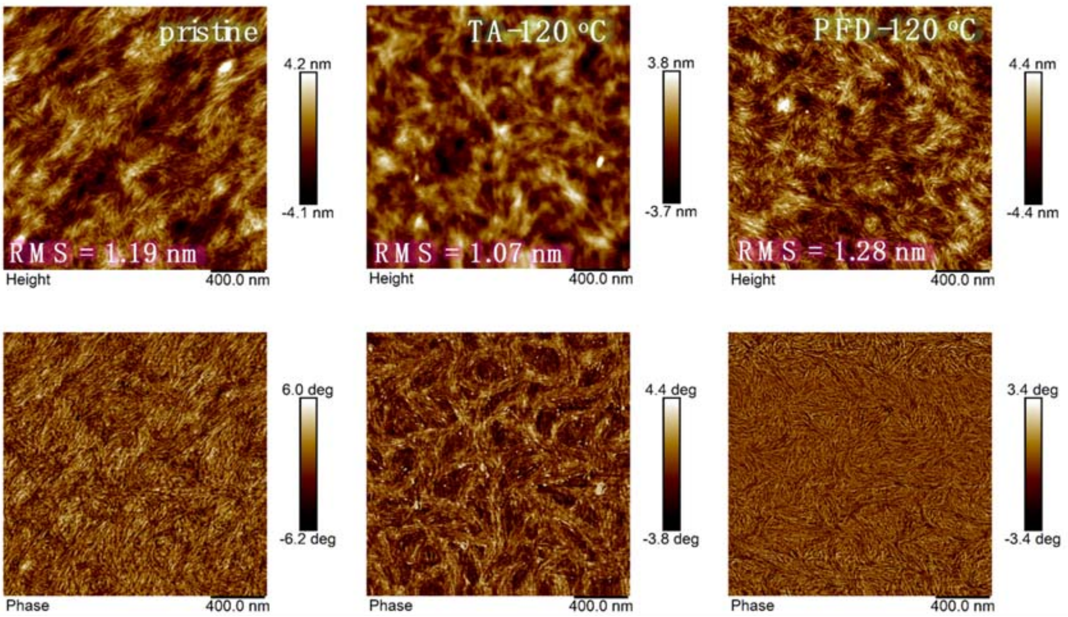

Supplementary Fig. 28 AFM height images and phase images of PTzBi-Si:N2200 films on the condition of pristine, TA-treated at 120 °C for 5 min, and PFD-treated at 120 °C for 1 min, respectively.

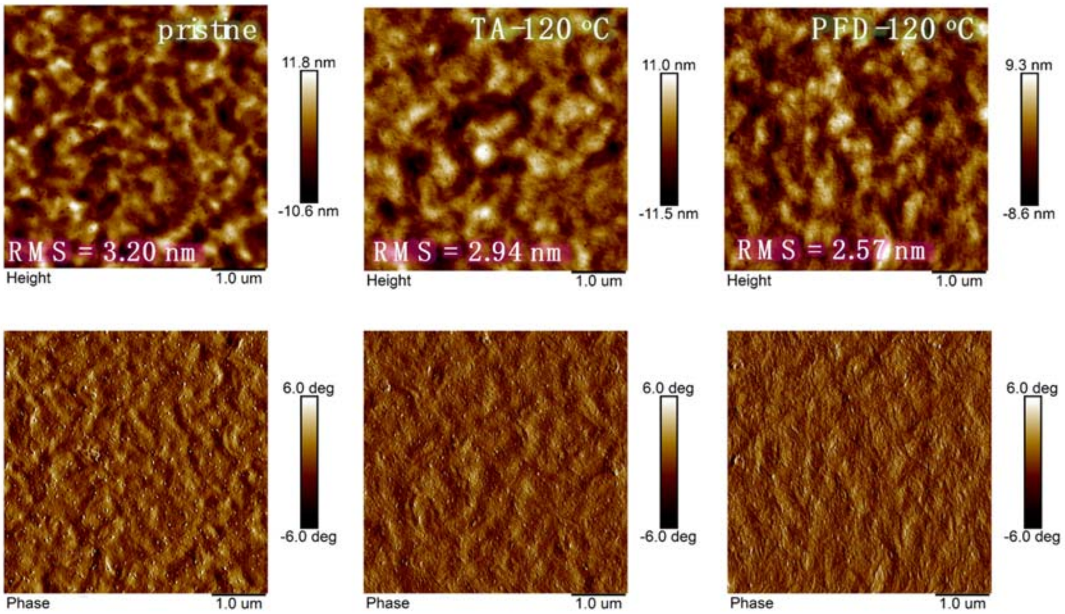

Supplementary Fig. 29 AFM height images and phase images of BTR-Cl:Y6 films on the condition of pristine, TA-treated at 120 °C for 5 min, and PFD-treated at 120 °C for 2 min, respectively.

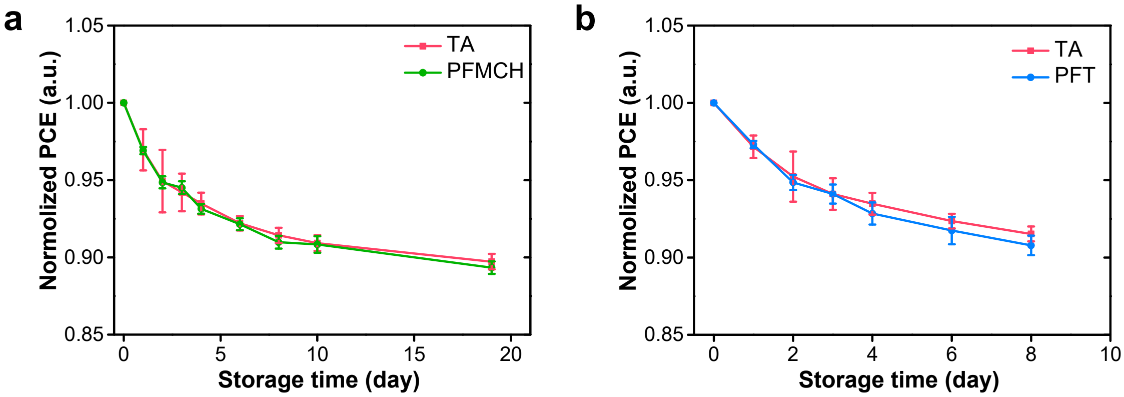

Supplementary Fig. 30 The stability test of PM6:Y6 devices as a function of storage time in a nitrogen-filled glove box without further encapsulation at temperature of 25-35 °C in dark. **a** TA devices and PFMCH-treated devices fabricated in one batch. **b** TA devices and PFT-treated devices fabricated in one batch. Error bars represent one standard deviation from the mean (n = 8).

3. Additional Tables

Supplementary Table 1 The photovoltaic parameters of PM6:Y6 devices by TA with different temperatures and time under the illumination of AM 1.5G, 100 mW cm^{-2}.

Time (min)	Temperature (°C)	V_{oc} (V)	J_{sc} (mA cm^{-2})	FF (%)	PCE[a] (%)
5	40	0.871	24.32	72.2	15.30 (15.10 ± 0.16)
5	50	0.867	24.50	72.4	15.38 (15.18 ± 0.15)
5	60	0.863	24.57	73.1	15.51 (15.28 ± 0.16)
5	70	0.858	25.03	73.1	15.70 (15.47 ± 0.17)
5	80	0.856	25.34	73.4	15.92 (15.68 ± 0.18)
5	90	0.854	25.51	73.3	15.96 (15.81 ± 0.11)
5	100	0.850	25.59	73.0	15.87 (15.78 ± 0.07)
5	110	0.846	25.40	73.1	15.71 (15.59 ± 0.09)
2	40	0.874	24.28	72.0	15.28 (15.00 ± 0.20)
2	50	0.869	24.48	72.2	15.36 (15.12 ± 0.16)
2	60	0.865	24.54	72.5	15.39 (15.11 ± 0.19)
2	70	0.860	24.69	72.9	15.48 (15.16 ± 0.21)
2	80	0.858	24.86	73.1	15.59 (15.31 ± 0.19)
2	90	0.856	25.04	73.3	15.71 (15.50 ± 0.14)
2	100	0.852	25.19	73.3	15.73 (15.52 ± 0.11)

[a] Average values with standard deviation in parentheses are statistically obtained from over 12 devices.

Supplementary Table 2 The photovoltaic parameters of PFD-treated PM6:Y6 devices with different temperatures and time under the illumination of AM 1.5G, 100 mW cm^{-2}.

Time (min)	Temperature (°C)	V_{oc} (V)	J_{sc} (mA cm^{-2})	FF (%)	PCE[a)] (%)
2	40	0.875	24.25	71.8	15.24 (15.06 ± 0.12)
2	50	0.872	24.41	72.1	15.34 (15.20 ± 0.10)
2	60	0.869	24.55	72.8	15.54 (15.38 ± 0.11)
2	70	0.866	25.00	73.1	15.83 (15.62 ± 0.15)
2	80	0.863	25.22	73.7	16.03 (15.85 ± 0.12)
2	90	0.861	25.30	74.0	16.12 (15.98 ± 0.09)
2	100	0.856	25.15	73.9	15.91 (15.91 ± 0.07)
1	90	0.864	25.12	73.6	15.97 (15.80 ± 0.13)
3	90	0.858	25.35	74.0	16.10 (15.89 ± 0.15)
4	90	0.855	25.34	73.8	15.99 (15.76 ± 0.17)

[a)] Average values with standard deviation in parentheses are statistically obtained from over 12 devices.

Supplementary Table 3 The photovoltaic parameters of PFMCH-treated PM6:Y6 devices with different temperatures and time under the illumination of AM 1.5G, 100 mW cm^{-2}.

Temperature (°C)	Time (min)	V_{oc} (V)	J_{sc} (mA cm^{-2})	FF (%)	PCE[a)] (%)
30	0.5	0.875	24.29	71.9	15.28 (15.01 ± 0.21)
30	1	0.874	24.26	71.6	15.23 (14.99 ± 0.18)
30	2	0.874	24.29	71.7	15.22 (14.93 ± 0.24)
40	0.5	0.873	24.31	72.1	15.31 (15.08 ± 0.18)
40	1	0.873	24.28	72.2	15.31 (15.03 ± 0.20)
40	2	0.873	24.33	71.9	15.27 (15.02 ± 0.19)
40	3	0.872	24.39	71.8	15.26 (14.96 ± 0.23)
45	0.5	0.873	24.29	72.5	15.38 (15.04 ± 0.24)
45	1	0.872	24.31	72.6	15.40 (15.05 ± 0.25)
45	2	0.872	24.44	72.8	15.51 (15.14 ± 0.25)
50	0.5	0.871	24.50	72.4	15.45 (15.09 ± 0.25)
50	1	0.869	24.57	73.1	15.59 (15.21 ± 0.27)
50	2	0.868	24.79	73.8	15.88 (15.57 ± 0.22)
50	3	0.867	24.92	74.2	16.03 (15.76 ± 0.19)
55	0.5	0.867	24.82	73.8	15.90 (15.43 ± 0.30)
55	1	0.866	25.19	74.9	16.34 (15.87 ± 0.30)
55	2	0.864	25.31	74.5	16.29 (16.00 ± 0.21)
60	0.5	0.864	25.02	74.1	16.02 (15.56 ± 0.28)
60	1	0.863	25.35	74.8	16.37 (16.09 ± 0.18)
60	2	0.861	25.37	74.2	16.21 (15.89 ± 0.23)
60	3	0.859	25.33	73.7	16.04 (15.76 ± 0.19)
70	0.5	0.860	25.26	74.3	16.14 (15.64 ± 0.32)
70	1	0.860	25.49	73.8	16.12 (15.90 ± 0.15)
70	2	0.858	25.42	73.6	16.05 (15.71 ± 0.23)

[a)] Average values with standard deviation in parentheses are statistically obtained from over 12 devices.

Supplementary Table 4 The photovoltaic parameters of control devices and PFMCH-treated devices with or without vacuum for fresh-made PM6:Y6 active layers under the illumination of AM 1.5G, 100 mW cm^{-2}.

vacuum [a]	post-treatment	V_{oc} (V)	J_{sc} (mA cm^{-2})	FF (%)	PCE[b] (%)
without	without	0.876	24.18	71.8	15.21 (15.08 ± 0.10)
without	PFMCH	0.863	25.35	74.8	16.37 (16.09 ± 0.18)
with	without	0.872	24.54	72.4	15.49 (15.33 ± 0.12)
with	PFMCH	0.865	24.77	72.9	15.62 (15.41 ± 0.15)

[a] Storing the fresh-made active layers in a vacuum chamber with pressure lower than 100 Pa for 12 hours; [b] Average values with standard deviation in parentheses are statistically obtained from over 12 devices.

Supplementary Table 5 The photovoltaic parameters of PFT-treated PM6:Y6 devices with different temperatures and time under the illumination of AM 1.5G, 100 mW cm^{-2}.

Time (s)	Temperature (°C)	V_{oc} (V)	J_{sc} (mA cm^{-2})	FF (%)	PCE[a] (%)
30	30	0.873	24.25	71.9	15.23 (15.03 ± 0.16)
30	40	0.869	24.67	72.0	15.44 (15.15 ± 0.19)
30	50	0.865	25.12	72.3	15.77 (15.48 ± 0.21)
30	60	0.860	25.63	73.2	16.14 (15.94 ± 0.15)
30	70	0.855	25.92	73.9	16.33 (16.19 ± 0.17)
30	80	0.849	26.09	74.6	16.52 (16.33 ± 0.16)
30	90	0.842	26.20	74.9	16.48 (16.22 ± 0.21)
30	100	0.830	26.06	74.4	16.10 (15.96 ± 0.13)
15	80	0.861	25.61	74.1	16.34 (16.02 ± 0.23)
45	80	0.837	25.94	75.1	16.31 (15.92 ± 0.27)
60	80	0.828	25.42	74.2	15.62 (15.31 ± 0.23)

[a] Average values with standard deviation in parentheses are statistically obtained from over 12 devices.

Supplementary Table 6 The carrier mobilities of PM6:Y6 devices with different post-treatments measured in single carrier diodes by fitting of SCLC model.

Post-treatment	μ_{hole}[a)](10^{-4} cm^2 V^{-1} s^{-1})	$\mu_{electron}$[a)] (10^{-4} cm^2 V^{-1} s^{-1})	$\mu_{electron}/\mu_{hole}$[b)]
pristine	3.34 (3.20 ± 0.11)	5.40 (5.15 ± 0.21)	1.62
TA	4.11 (3.91 ± 0.15)	6.73 (6.41 ± 0.26)	1.64
PFD	4.73 (4.53 ± 0.17)	7.01 (6.61 ± 0.28)	1.48
PFMCH	3.59 (3.45 ± 0.12)	5.63 (5.36 ± 0.22)	1.57
PFT	6.72 (6.41 ± 0.25)	7.35 (6.91 ± 0.35)	1.09

[a)] Average values with standard deviation in parentheses are statistically obtained from over 8 devices; [b)] The ratio of maximum value.

Supplementary Table 7 Characteristic length scale of packing phenomenon in PM6:Y6 blend films with different post-treatments.

Post-treatment	π-π stacking in OOP (010)				Lamellar packing in IP (100)	
	Peak location ($Å^{-1}$)	d-spacing (Å)	FWHM[a)] ($Å^{-1}$)	CCL[b)] (Å)	Peak location ($Å^{-1}$)	CCL[b)] (Å)
pristine	1.73	3.63	0.291	19.4	0.293	21.4
TA	1.73	3.63	0.291	19.4	0.294	21.4
PFD	1.73	3.63	0.291	19.4	0.294	21.4
PFMCH	1.73	3.63	0.293	19.3	0.293	21.4
PFT	1.75	3.59	0.279	20.3	0.294	21.4

[a)] FWHM represents full-width at half maximum; [b)] CCL represents crystal coherence length.

Supplementary Table 8 The photovoltaic parameters of PM6:IT4F devices under the illumination of AM 1.5G, 100 mW cm^{-2}. CF:DIO (99.5:0.5, v/v) as processing solvent.

Post-treatment	Temperature (°C)	V_{oc} (V)	J_{sc} (mA cm^{-2})	FF (%)	PCE (%)
w/o	Non.	0.86	19.93	74.7	12.81
TA [a]	90	0.85	19.84	75.9	12.80
	100	0.85	19.82	76.0	12.81
PFT	80 [b]	0.86	19.90	75.8	12.97
	80 [c]	0.86	19.91	76.5	13.10

[a] 5 min. [b] 0.5 min. [c] 0.75 min.

Supplementary Table 9 The photovoltaic parameters of PTB7-Th:IEICO-4F devices under the illumination of AM 1.5G, 100 mW cm^{-2}. CF:CN (96.5:3.5, v/v) as processing solvent.

Post-treatment	Temperature (°C)	V_{oc} (V)	J_{sc} (mA cm^{-2})	FF (%)	PCE (%)
w/o	Non.	0.71	23.75	66.6	11.22
TA	90 [a]	0.70	23.69	67.9	11.26
	100 [a]	0.70	23.84	67.7	11.29
	100 [b]	0.70	23.43	67.9	11.14
	110 [a]	0.69	23.77	67.5	11.07
PFT [c]	60	0.72	23.28	70.4	11.80
	70	0.71	24.15	71.3	12.23
	80	0.71	23.57	71.0	11.88

[a] 5 min. [b] 10 min. [c] 0.5 min.

Supplementary Table 10 The photovoltaic parameters of PFD-treated P3HT:o-IDTBR devices with different temperatures and time under the illumination of AM 1.5G, 100 mW cm^{-2}. CF:CN (99.5:0.5, v/v) as processing solvent.

Post-treatment	Temperature (°C)	V_{oc} (V)	J_{sc} (mA cm^{-2})	FF (%)	PCE (%)
w/o	Non.	0.76	10.45	45.4	3.60
TA [a]	40	0.77	10.28	46.9	3.71
	50	0.77	10.60	44.6	3.64
	60	0.78	10.08	48.1	3.78
	65	0.77	10.14	49.3	3.85
	80	0.76	10.62	50.8	4.10
	100	0.75	11.11	54.2	4.52
	110	0.75	13.24	60.0	5.96
	120	0.75	12.84	59.3	5.63
PFD [b]	40	0.76	10.45	45.5	3.61
	50	0.77	10.43	45.8	3.68
	60	0.78	10.09	46.9	3.69
	65	0.77	9.49	50.9	3.72
	80	0.77	10.07	53.4	4.12
	100	0.75	11.25	57.1	4.75
	110	0.75	13.25	61.9	6.15
	120	0.75	12.31	62.3	5.75
PFMCH [c]	40	0.76	10.52	45.8	3.66
	50	0.76	10.60	46.1	3.75
	55	0.79	10.12	49.4	3.95
	60	0.78	10.58	56.5	4.66
	65	0.79	10.94	56.8	4.91
	70	0.78	11.02	55.7	4.79

[a] 10 min. [b] 3 min. [c] 1 min.

Supplementary Table 11 The photovoltaic parameters of PFD-treated P3HT:o-IDTBR devices with different temperatures and time under the illumination of AM 1.5G, 100 mW cm^{-2}. CB as processing solvent.

Post-treatment	Temperature (°C)	V_{oc} (V)	J_{sc} (mA cm^{-2})	FF (%)	PCE (%)
w/o	Non.	0.69	10.43	51.4	3.67
TA [a]	80	0.71	10.32	61.7	4.48
	90	0.71	11.32	62.0	4.98
	100	0.73	11.27	64.1	5.27
	110	0.74	12.71	64.2	6.04
	120	0.74	12.11	67.5	6.05
	130	0.73	10.72	66.7	5.22
PFD [b]	80	0.72	10.80	67.3	5.23
	90	0.72	12.03	65.1	5.64
	100	0.74	11.61	67.8	5.82
	110	0.73	12.61	71.3	6.56
	120	0.73	12.01	71.0	6.23
	130	0.72	11.85	69.2	5.91

[a] 10 min. [b] 1 min.

Supplementary Table 12 The photovoltaic parameters of CB processed P3HT:o-IDTBR devices with or without vacuum process to fresh-made active layer films.

	post-treatment	V_{oc} (V)	J_{sc} (mA cm^{-2})	FF (%)	PCE (%)
without vacuum	pristine	0.69	10.43	51.4	3.67
	TA for 10 min	0.74	12.11	67.5	6.05
	PFD for 1 min	0.73	12.61	71.3	6.56
with vacuum	pristine	0.70	9.67	56.7	3.84
	TA for 10 min	0.73	12.45	65.4	5.94
	PFD for 1 min	0.72	12.07	69.4	6.03

Supplementary Table 13 The photovoltaic parameters of PTzBi-Si:N2200 devices under the illumination of AM 1.5G, 100 mW cm^{-2}. MeTHF as processing solvent.

Post-treatment	Temperature (°C)	V_{oc} (V)	J_{sc} (mA cm^{-2})	FF (%)	PCE (%)
w/o	Non.	0.89	14.21	56.9	7.20
TA [a]	90	0.88	14.42	61.5	7.80
	100	0.88	14.68	63.6	8.22
	120	0.88	14.92	69.4	9.11
	130	0.88	14.57	70.9	9.09
PFD [b]	90	0.88	13.52	71.8	8.54
	100	0.88	13.61	73.2	8.77
	110	0.88	14.63	71.8	9.24
	120	0.88	14.49	73.7	9.40
	130	0.88	14.44	73.7	9.37

[a] 5 min. [b] 1 min.

Supplementary Table 14 The photovoltaic parameters of PFD-treated BTR-Cl:Y6 devices with different temperatures and time under the illumination of AM 1.5G, 100 mW cm^{-2}. CF as processing solvent.

Post-treatment	Temperature (°C)	V_{oc} (V)	J_{sc} (mA cm^{-2})	FF (%)	PCE (%)
w/o	Non.	0.93	4.52	22.8	0.96
TA [a]	90	0.88	20.81	58.8	10.76
	100	0.85	22.42	63.6	12.12
	110	0.85	22.97	64.7	12.63
	120	0.85	23.13	67.9	13.36
	130	0.84	22.96	69.2	13.35
PFD [b]	90	0.88	21.53	56.1	10.62
	100	0.86	22.21	61.9	11.95
	110	0.85	22.29	68.0	12.89
	120	0.85	24.15	68.1	13.98
	130	0.84	23.02	69.9	13.52

[a] 5 min. [b] 2 min.

Supplementary Table 15 The photovoltaic parameters of PFD-treated BTR-Cl:Y6 or PTzBi-Si:N2200 devices with or without vacuum process to fresh-made active layer films.

active layer		V_{oc} (V)	J_{sc} (mA cm^{-2})	FF (%)	PCE (%)
BTR-Cl:Y6 [a]	without vacuum	0.85	23.70	68.2	13.73
	with vacuum	0.85	23.47	68.3	13.63
PTzBi-Si:N2200 [b]	without vacuum	0.88	14.49	73.7	9.40
	with vacuum	0.88	14.51	72.8	9.29

[a] CF as processing solvent and PFD-treated at 120 °C for 2 min. [b] MeTHF as processing solvent and PFD-treated at 120 °C for 1 min..

Supplementary Table 16 The absorption peak of Y6 in PM6:Y6 blend films with different post-treatments.

Temperature (°C)	TA (nm)	PFD (nm)	PFMCH (nm)	PFT (nm)
pristine	800	800	800	800
30	N.A.	N.A.	801	801
40	803	802	803	807
45	N.A.	N.A.	803	N.A.
50	804	803	803	811
55	N.A.	N.A.	804	N.A.
60	806	804	805	815
70	807	808	806	819
80	809	811	N.A.	821
90	810	814	N.A.	822
100	811	814	N.A.	822
