# Supplementary material for: Universal and versatile morphology engineering via hot fluorous solvent soaking for organic bulk heterojunction
Source: Nat Commun. 2020 Nov 4;11:5585. doi: 10.1038/s41467-020-19429-x (PMC7642440; doi:10.1038/s41467-020-19429-x)
Supplement: Supplementary file 2 — Reporting Summary [file 41467_2020_19429_MOESM2_ESM.pdf]

nature research

Corresponding author(s): *Double-blind peer review submissions: write DBPR and your manuscript number here instead of author names.*

# Solar Cells Reporting Summary

Nature Research wishes to improve the reproducibility of the work that we publish. This form is intended for publication with all accepted papers reporting the characterization of photovoltaic devices and provides structure for consistency and transparency in reporting. Some list items might not apply to an individual manuscript, but all fields must be completed for clarity.

For further information on Nature Research policies, including our data availability policy, see Authors & Referees.

## Experimental design

**Please check: are the following details reported in the manuscript?**

1. Dimensions

| Item | Yes | No | Details |
|---|---|---|---|
| Area of the tested solar cells | ☒ | ☐ | Method section. |
| Method used to determine the device area | ☒ | ☐ | Method section. |

2. Current-voltage characterization

| Item | Yes | No | Details |
|---|---|---|---|
| Current density-voltage (J-V) plots in both forward and backward direction | ☐ | ☒ | J-V plots in both foward and backard direction of organic solar are known identical, so only forward ones are supplied. |
| Voltage scan conditions *For instance: scan direction, speed, dwell times* | ☒ | ☐ | Method section. |
| Test environment *For instance: characterization temperature, in air or in glove box* | ☒ | ☐ | Method section. |
| Protocol for preconditioning of the device before its characterization | ☒ | ☐ | Method section. |
| Stability of the J-V characteristic *Verified with time evolution of the maximum power point or with the photocurrent at maximum power point; see ref. 7 for details.* | ☒ | ☐ | Discussion in main text and figure in SI. |

3. Hysteresis or any other unusual behaviour

| Item | Yes | No | Details |
|---|---|---|---|
| Description of the unusual behaviour observed during the characterization | ☐ | ☒ | Organic solar are known without hysteresis behaviour. |
| Related experimental data | ☐ | ☒ | Organic solar are known without hysteresis behaviour. |

4. Efficiency

| Item | Yes | No | Details |
|---|---|---|---|
| External quantum efficiency (EQE) or incident photons to current efficiency (IPCE) | ☒ | ☐ | Figures in main text and SI. |
| A comparison between the integrated response under the standard reference spectrum and the response measure under the simulator | ☒ | ☐ | Figures and discussion in main text. |
| For tandem solar cells, the bias illumination and bias voltage used for each subcell | ☐ | ☒ | No tandem solar cell is reported in this work. |

5. Calibration

| Item | Yes | No | Details |
|---|---|---|---|
| Light source and reference cell or sensor used for the characterization | ☒ | ☐ | Method section. |
| Confirmation that the reference cell was calibrated and certified | ☒ | ☐ | Method section. |

| | | |
|---|---|---|
| Calculation of spectral mismatch between the reference cell and the devices under test | ☒ Yes ☐ No | Method section. |

6. Mask/aperture

| | | |
|---|---|---|
| Size of the mask/aperture used during testing | ☐ Yes ☒ No | No mask/aperture was used during testing. The device area was defined by the intersection of anode and cathode and calibrated by microscope. |
| Variation of the measured short-circuit current density with the mask/aperture area | ☐ Yes ☒ No | No mask/aperture was used during testing. |

7. Performance certification

| | | |
|---|---|---|
| Identity of the independent certification laboratory that confirmed the photovoltaic performance | ☐ Yes ☒ No | The tests have been performed under standard condition with certified equipments, so it is not necessary of independent certification. |
| A copy of any certificate(s) *Provide in Supplementary Information* | ☐ Yes ☒ No | The tests have been performed under standard condition with certified equipments, so it is not necessary of independent certification. |

8. Statistics

| | | |
|---|---|---|
| Number of solar cells tested | ☒ Yes ☐ No | Figures and tables in main text and SI. |
| Statistical analysis of the device performance | ☒ Yes ☐ No | Figures, tables and discussion in main text and SI. |

9. Long-term stability analysis

| | | |
|---|---|---|
| Type of analysis, bias conditions and environmental conditions *For instance: illumination type, temperature, atmosphere humidity, encapsulation method, preconditioning temperature* | ☒ Yes ☐ No | Discussion in main text and figure in SI. |
